# Supplementary material for: MIA40 suppresses cell death induced by apoptosis-inducing factor 1
Source: EMBO Rep. 2025 Mar 7;26(7):1835–62. doi: 10.1038/s44319-025-00406-8 (PMC11976965; doi:10.1038/s44319-025-00406-8)
Supplement: Supplementary file 5 — Source data Fig. 1 [file 44319_2025_406_MOESM5_ESM.zip › Figure 1/Figure 1A/FCS Accessory subunit KOs/READ ME.docx]

FACS Accessory subunit KOs.

The file is labeled by the name of the cell lines plus treatment.

Control vehicle = DMSO 0.2%.

Ssporin casp = cell death induced by Staurosporine in presence of caspase inhibitor.

FITC = Annexin V stained cells for gating.

PI = Propidium Iodide stained cells for gating.

FITC + PI = Annexin V + Propidium Iodide stained cells for gating.

Control unstained = cells without FITC or PI for gating.
