# Supplementary material for: MIA40 suppresses cell death induced by apoptosis-inducing factor 1
Source: EMBO Rep. 2025 Mar 7;26(7):1835–62. doi: 10.1038/s44319-025-00406-8 (PMC11976965; doi:10.1038/s44319-025-00406-8)
Supplement: Supplementary file 5 — Source data Fig. 1 [file 44319_2025_406_MOESM5_ESM.zip › Figure 1/Figure 1D/READ ME.docx]

READ ME

Lines 1 to 4 = Load

Lines 5 to 6 = Eluate

Lines 7 to 12 = Unbound

1 = HEK293T transfected with empty vector

2 = NDUFA13-KO transfected with empty vector

3 = HEK293T transfected with MIA40_FLAG_

4 = NDUFA13-KO transfected with MIA40_FLAG_

5 = HEK293T transfected with empty vector

6 = NDUFA13-KO transfected with empty vector

7 = HEK293T transfected with MIA40_FLAG_

8 = NDUFA13-KO transfected with MIA40_FLAG_

9 = HEK293T transfected with empty vector

10 = NDUFA13-KO transfected with empty vector

11 = HEK293T transfected with MIA40_FLAG_

12 = NDUFA13-KO transfected with MIA40_FLAG_
