# Supplementary figures and images for: MIA40 suppresses cell death induced by apoptosis-inducing factor 1
Source: EMBO Rep. 2025 Mar 7;26(7):1835–62. doi: 10.1038/s44319-025-00406-8 (PMC11976965; doi:10.1038/s44319-025-00406-8)

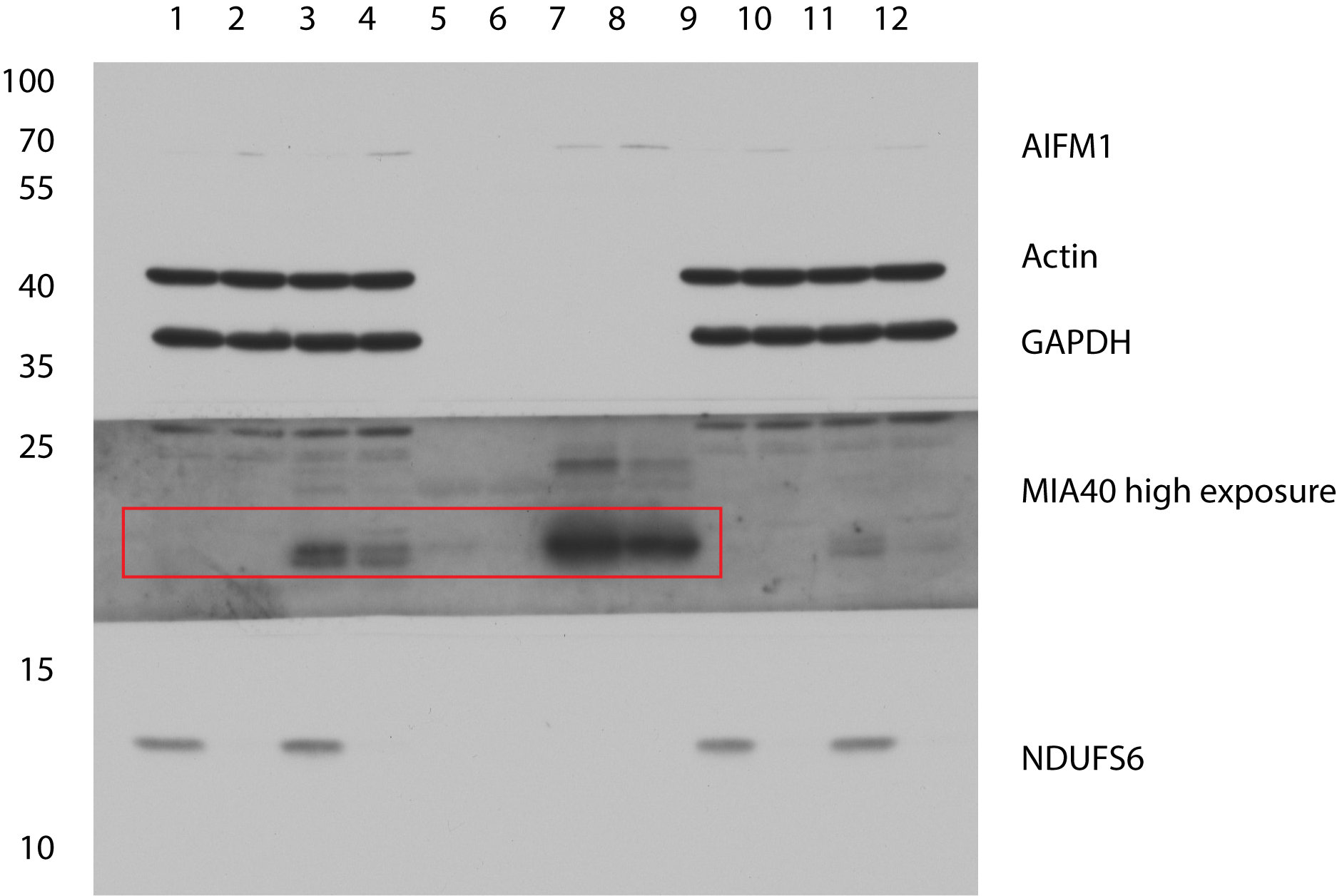

Supplement: Supplementary file 5 — Source data Fig. 1 [file 44319_2025_406_MOESM5_ESM.zip › Figure 1/Figure 1D/Original western with area cut/MIA40 high exposure original blottig.tif]

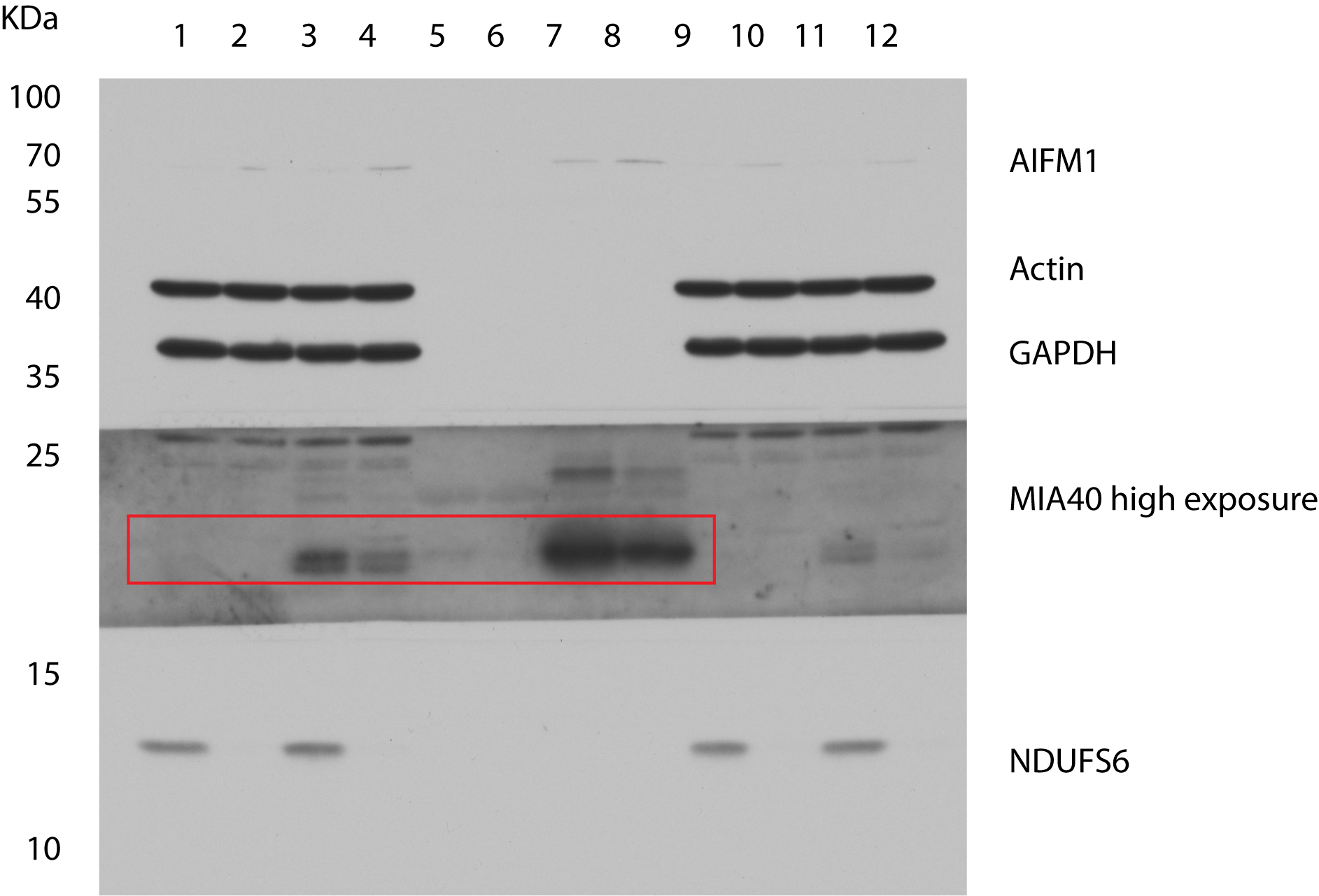

Supplement: Supplementary file 5 — Source data Fig. 1 [file 44319_2025_406_MOESM5_ESM.zip › Figure 1/Figure 1D/Original western with area cut/MIA40 high exposure original western.tif]

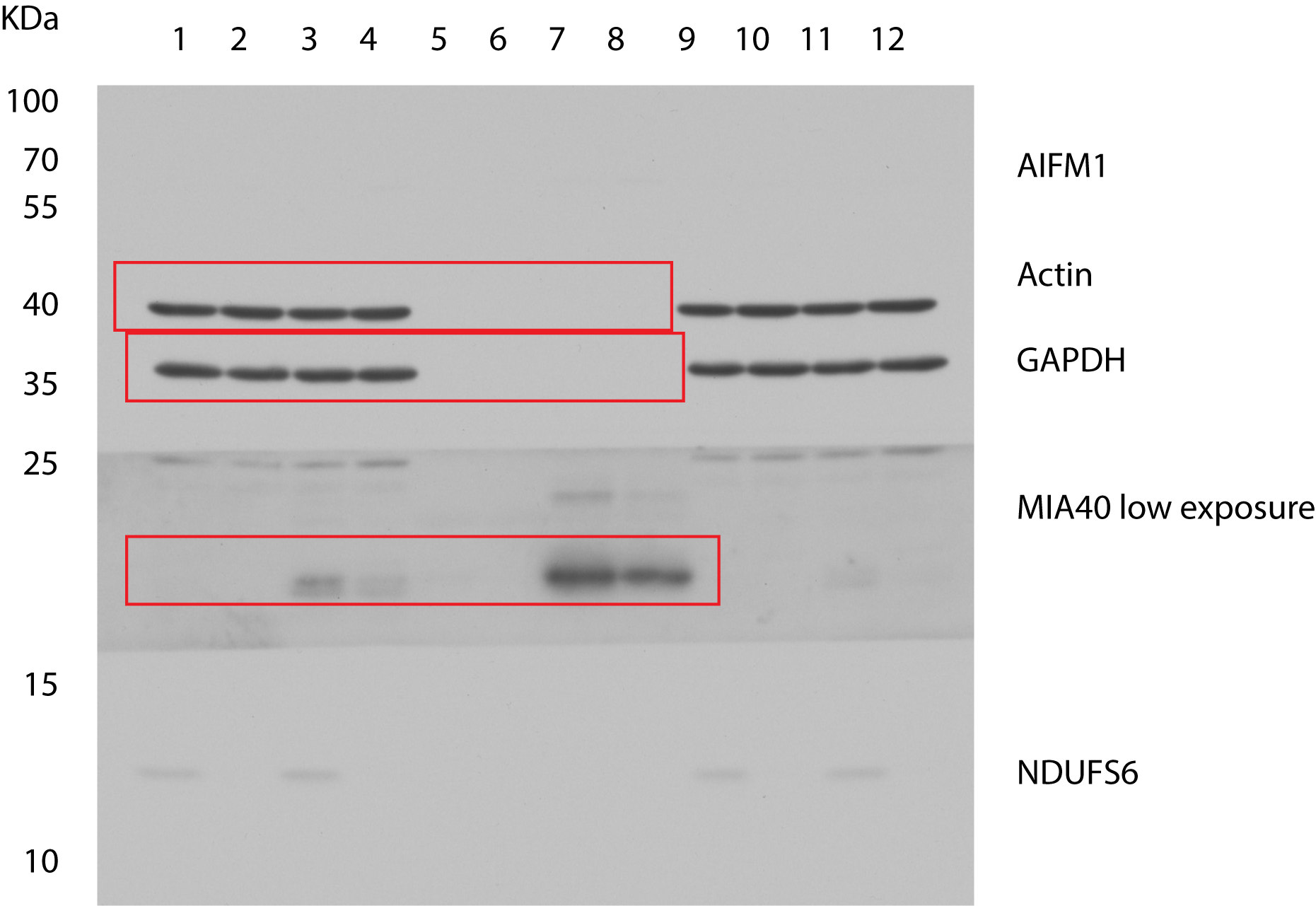

Supplement: Supplementary file 5 — Source data Fig. 1 [file 44319_2025_406_MOESM5_ESM.zip › Figure 1/Figure 1D/Original western with area cut/MIA40 low exposure, Actin and GAPDH original western.tif]

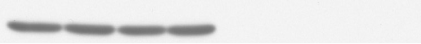

Supplement: Supplementary file 5 — Source data Fig. 1 [file 44319_2025_406_MOESM5_ESM.zip › Figure 1/Figure 1D/Wester each antibody/Actin cut.tif]

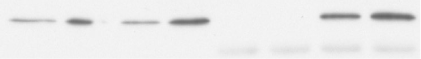

Supplement: Supplementary file 5 — Source data Fig. 1 [file 44319_2025_406_MOESM5_ESM.zip › Figure 1/Figure 1D/Wester each antibody/AIFM1 cut.tif]

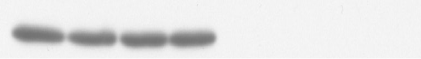

Supplement: Supplementary file 5 — Source data Fig. 1 [file 44319_2025_406_MOESM5_ESM.zip › Figure 1/Figure 1D/Wester each antibody/GAPDH cut.tif]

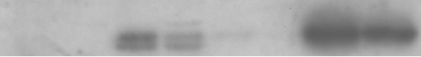

Supplement: Supplementary file 5 — Source data Fig. 1 [file 44319_2025_406_MOESM5_ESM.zip › Figure 1/Figure 1D/Wester each antibody/MIA40 high exposure cut.tif]

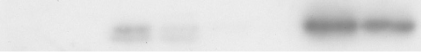

Supplement: Supplementary file 5 — Source data Fig. 1 [file 44319_2025_406_MOESM5_ESM.zip › Figure 1/Figure 1D/Wester each antibody/MIA40 low exposure cut.tif]

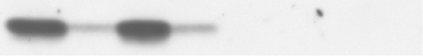

Supplement: Supplementary file 5 — Source data Fig. 1 [file 44319_2025_406_MOESM5_ESM.zip › Figure 1/Figure 1D/Wester each antibody/NDUFS6 cut.tif]

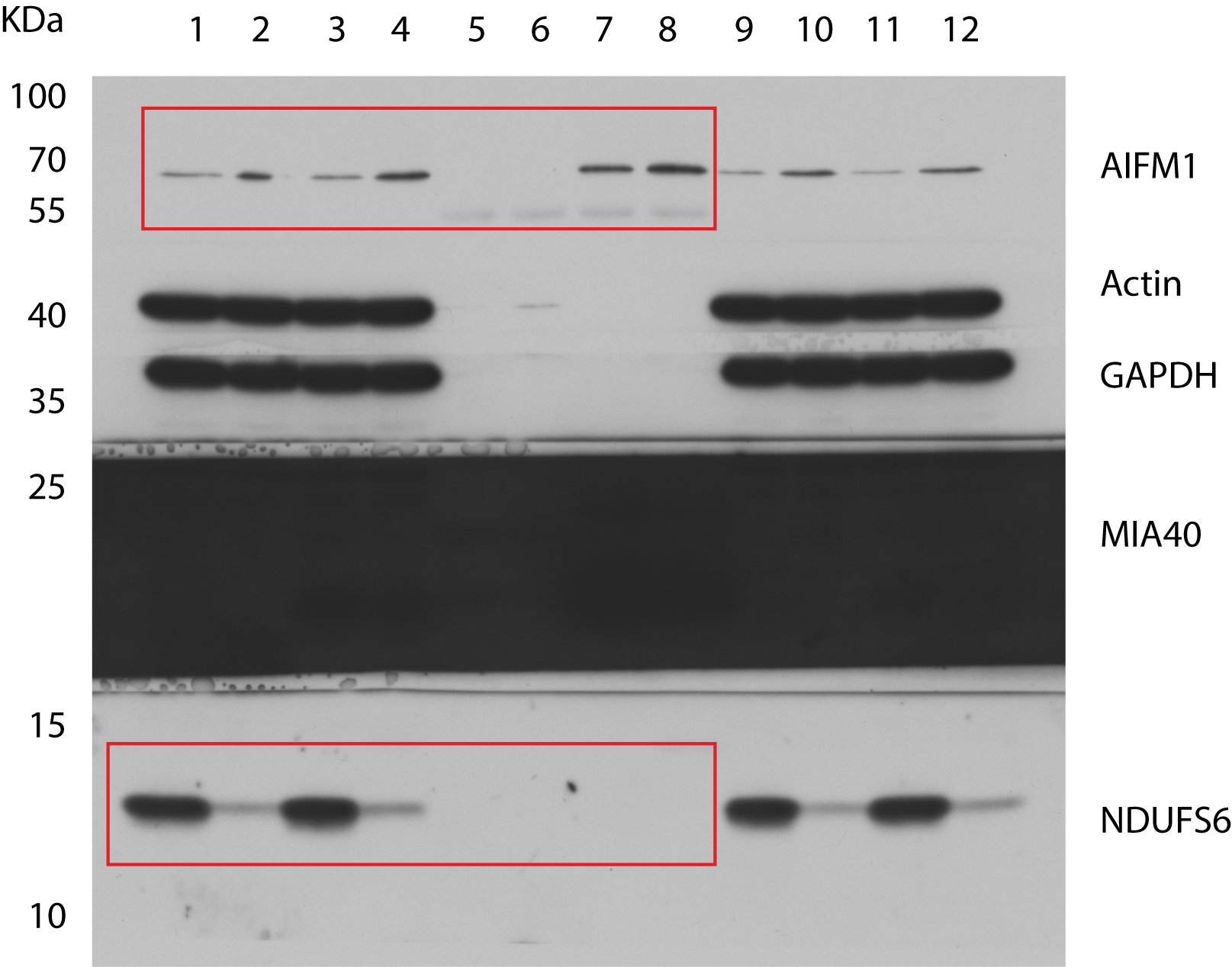

Supplement: Supplementary file 5 — Source data Fig. 1 [file 44319_2025_406_MOESM5_ESM.zip › Figure 1/Figure 1D/Western orignal/AIFM1 and NDUFS6 original western.tif]

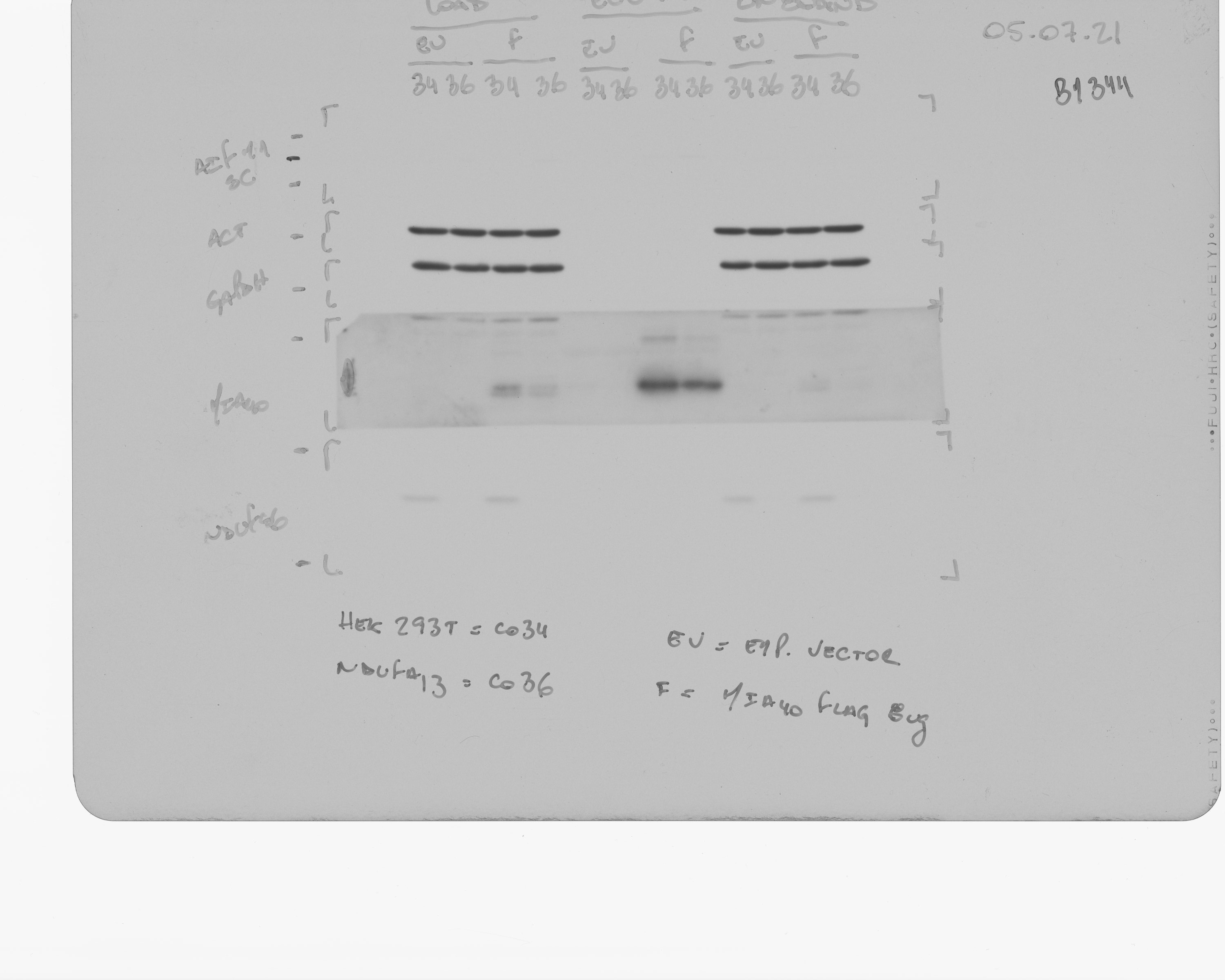

Supplement: Supplementary file 5 — Source data Fig. 1 [file 44319_2025_406_MOESM5_ESM.zip › Figure 1/Figure 1D/Western orignal/NDUFA13KO023.jpg]

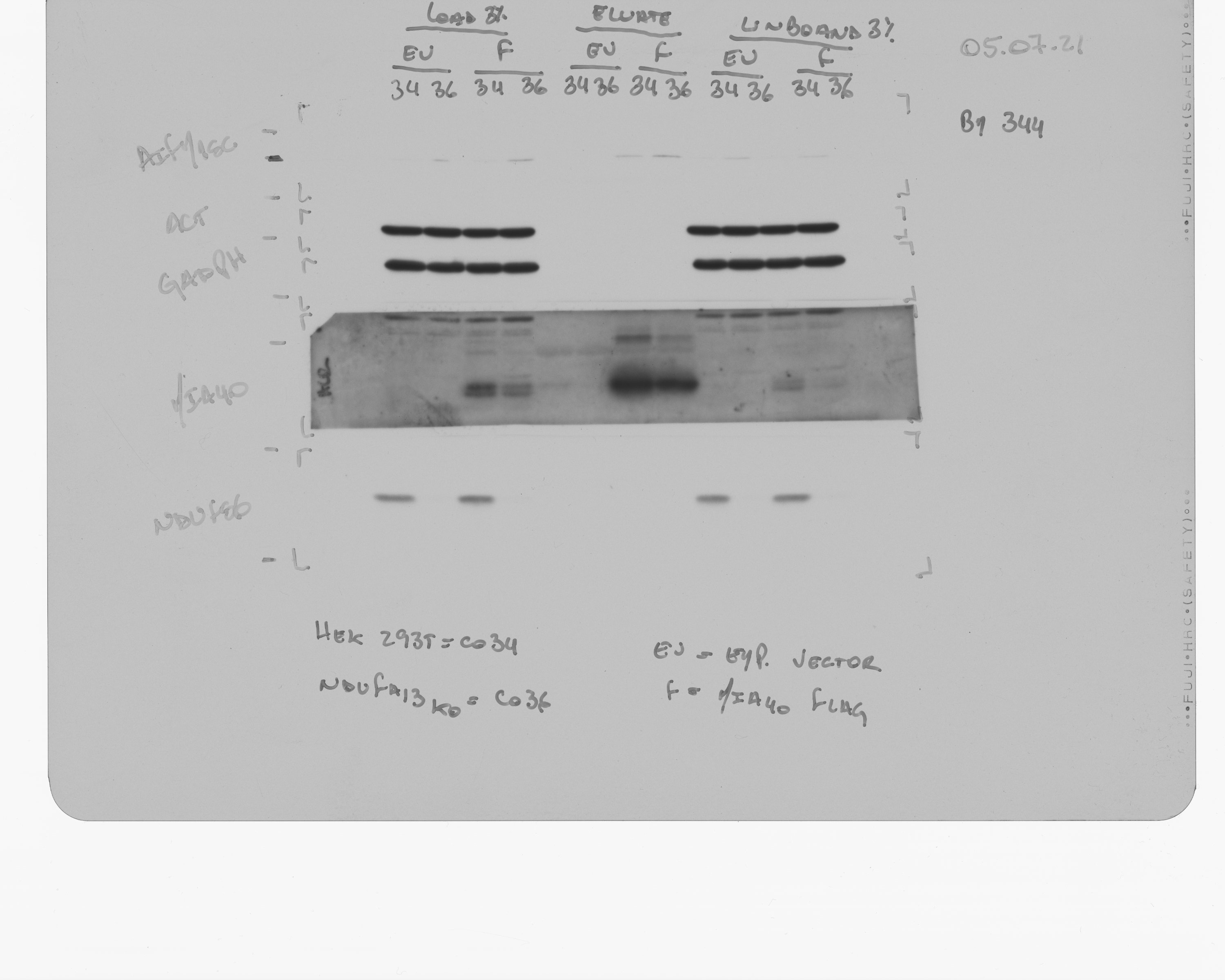

Supplement: Supplementary file 5 — Source data Fig. 1 [file 44319_2025_406_MOESM5_ESM.zip › Figure 1/Figure 1D/Western orignal/NDUFA13KO024.jpg]

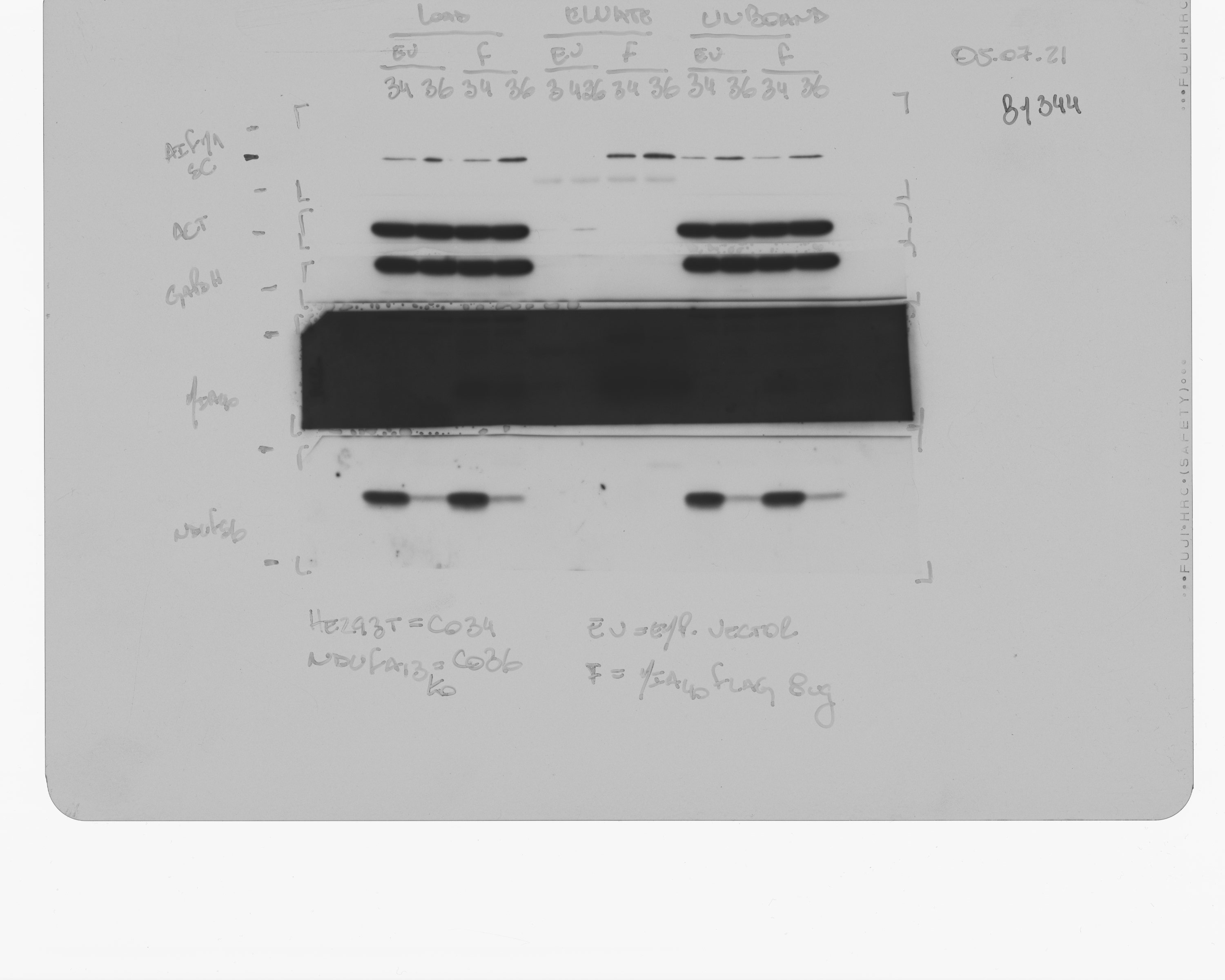

Supplement: Supplementary file 5 — Source data Fig. 1 [file 44319_2025_406_MOESM5_ESM.zip › Figure 1/Figure 1D/Western orignal/NDUFA13KO025.jpg]

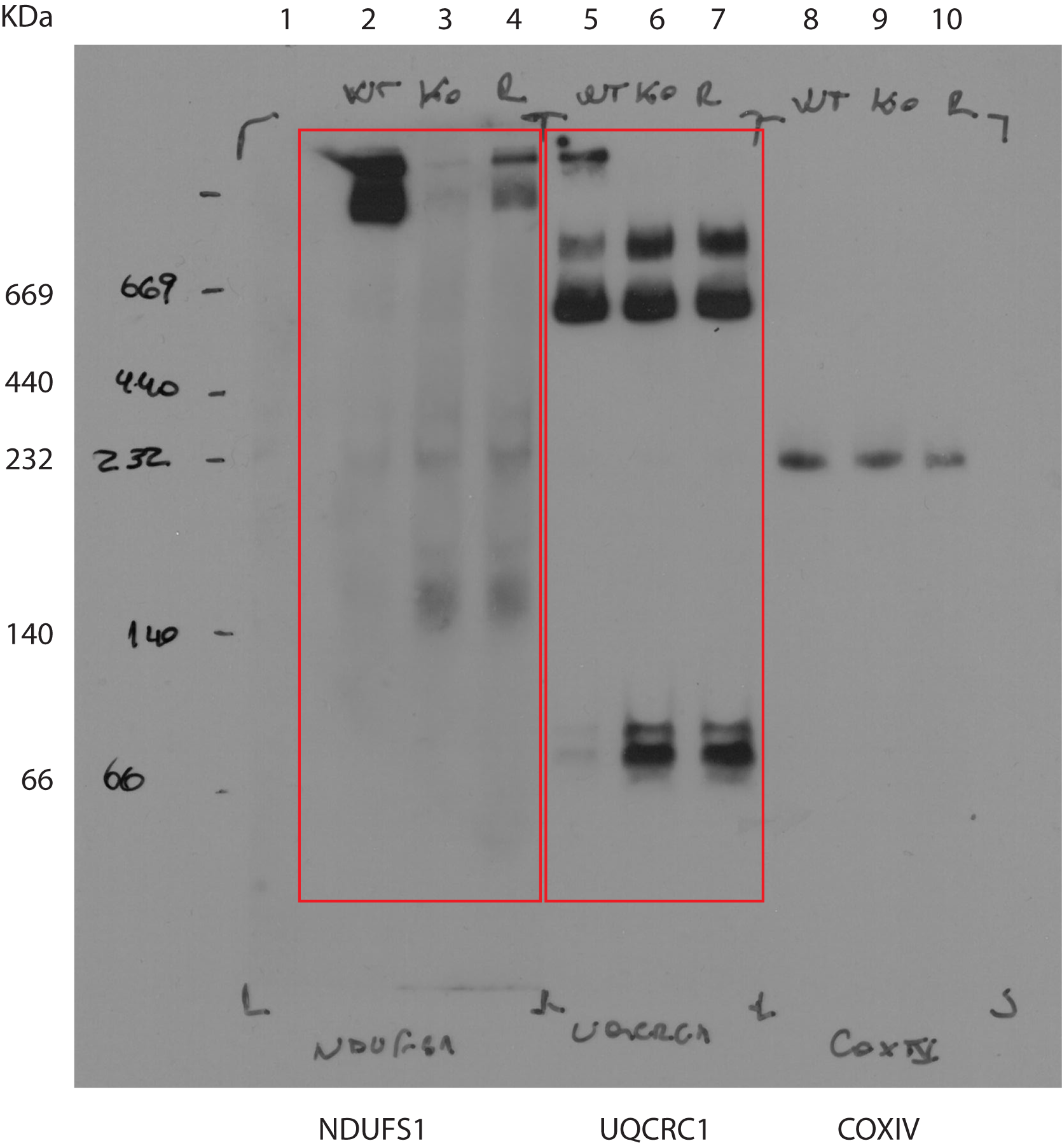

Supplement: Supplementary file 6 — Source data Fig. 2 [file 44319_2025_406_MOESM6_ESM.zip › Figure 2/Figure 2C/Rescue 24 h/Original western with area cut/Rescue 24h Blue native.tif]

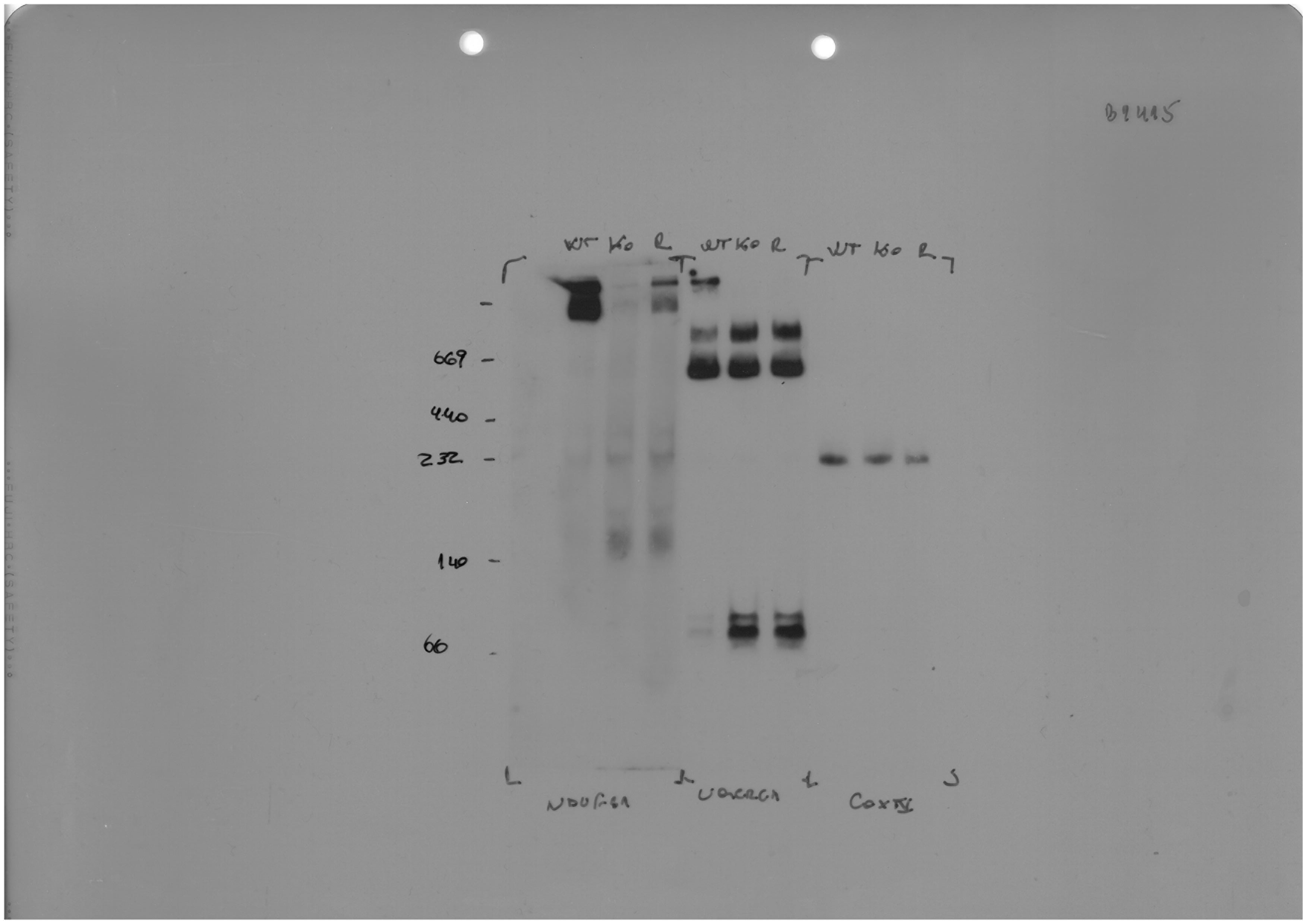

Supplement: Supplementary file 6 — Source data Fig. 2 [file 44319_2025_406_MOESM6_ESM.zip › Figure 2/Figure 2C/Rescue 24 h/Original western/24 h rescue.tif]

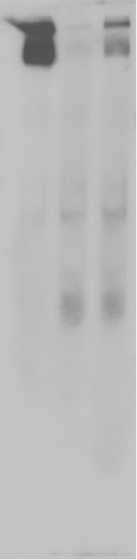

Supplement: Supplementary file 6 — Source data Fig. 2 [file 44319_2025_406_MOESM6_ESM.zip › Figure 2/Figure 2C/Rescue 24 h/Western cut for each antibody/NDUFS1 rescue 24 h cut.tif]

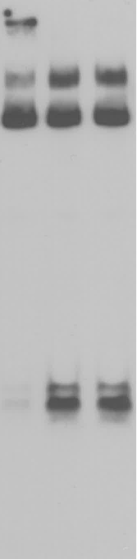

Supplement: Supplementary file 6 — Source data Fig. 2 [file 44319_2025_406_MOESM6_ESM.zip › Figure 2/Figure 2C/Rescue 24 h/Western cut for each antibody/UQCRC1 rescue 24 h cut.tif]

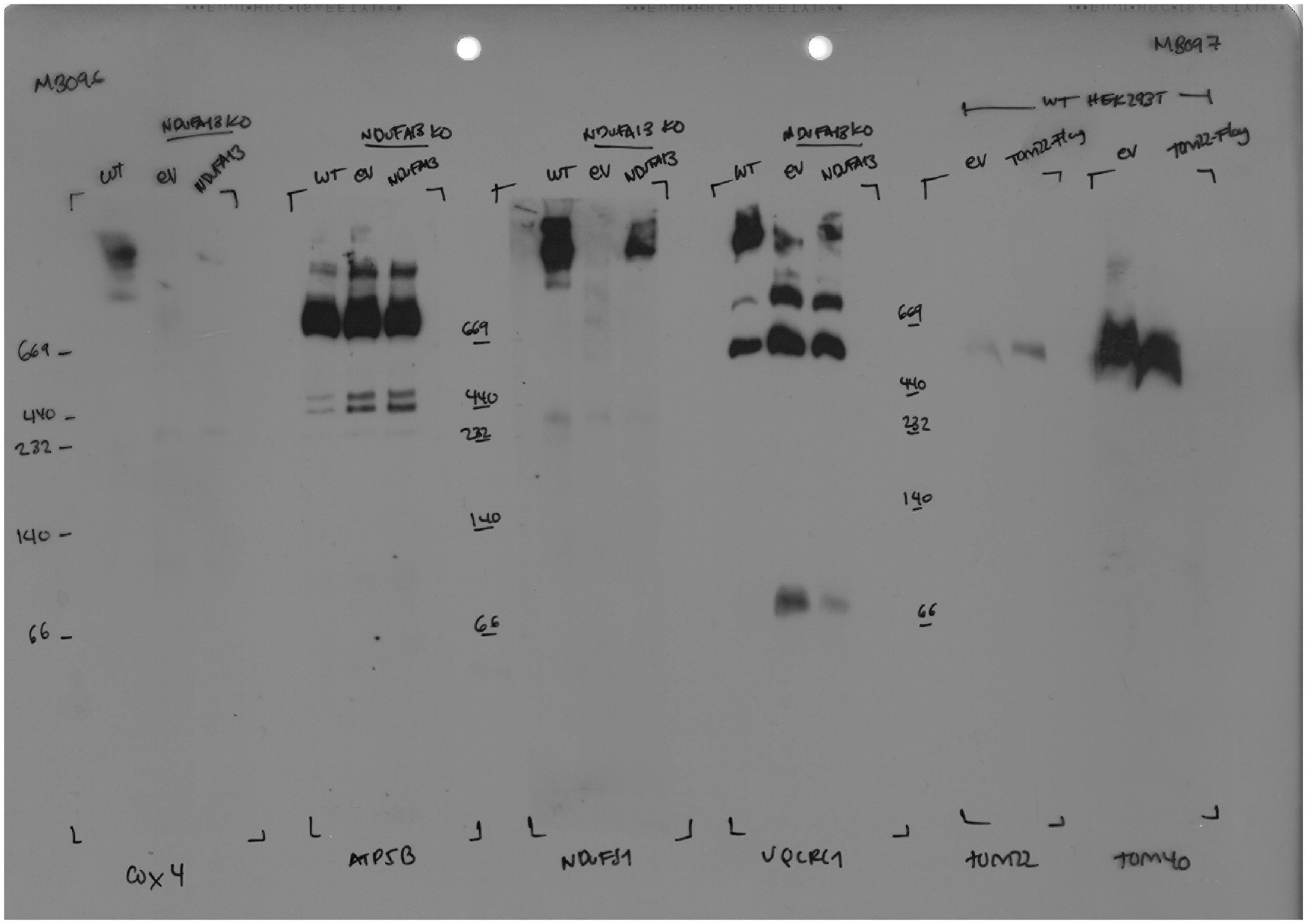

Supplement: Supplementary file 6 — Source data Fig. 2 [file 44319_2025_406_MOESM6_ESM.zip › Figure 2/Figure 2C/Rescue 72 h/Original Western/72 h rescue.tif]

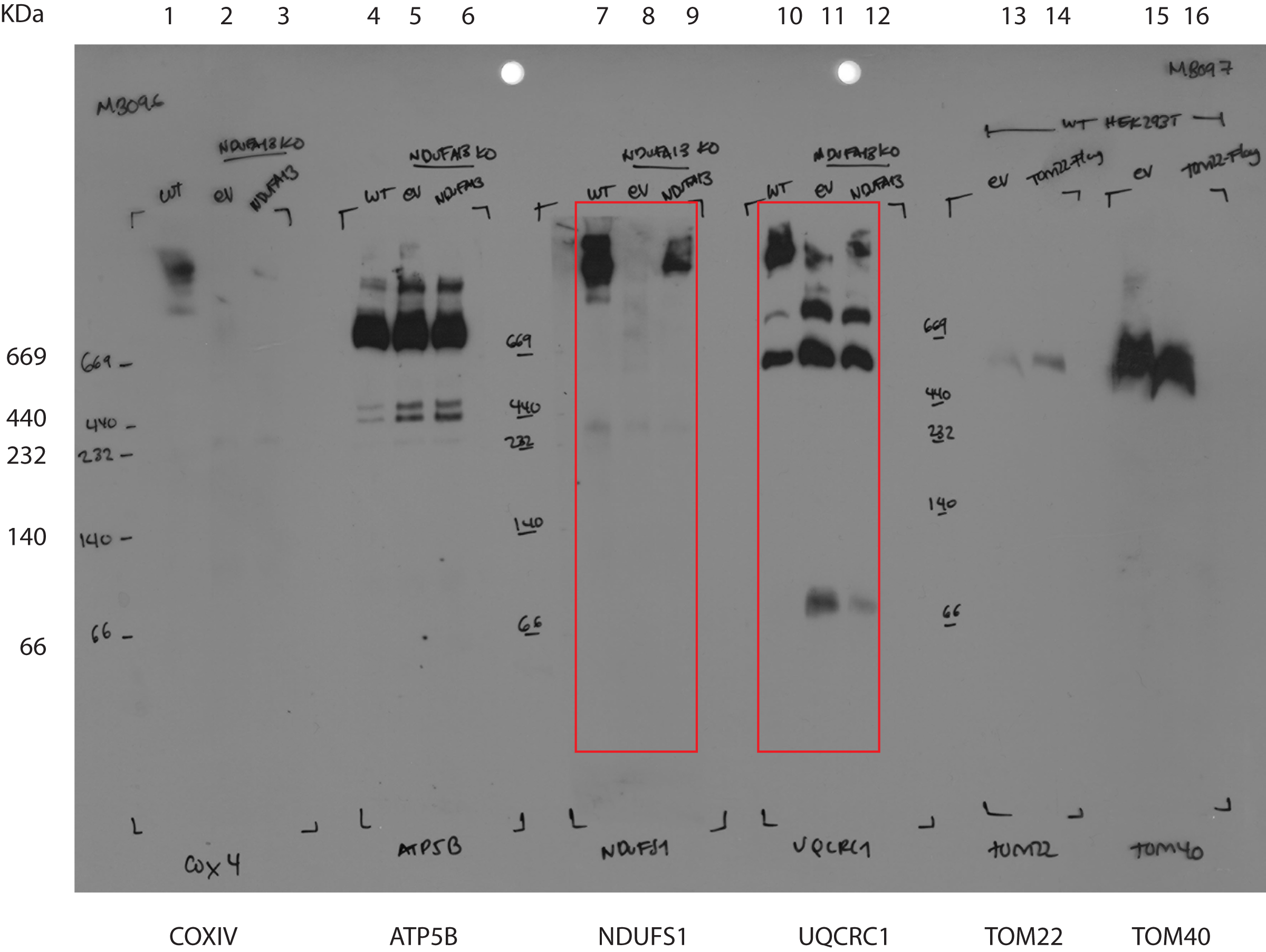

Supplement: Supplementary file 6 — Source data Fig. 2 [file 44319_2025_406_MOESM6_ESM.zip › Figure 2/Figure 2C/Rescue 72 h/Westen of each area cut/Rescue 72h Blue native.tif]

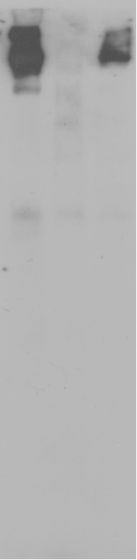

Supplement: Supplementary file 6 — Source data Fig. 2 [file 44319_2025_406_MOESM6_ESM.zip › Figure 2/Figure 2C/Rescue 72 h/Western of each antibody/NDUFS1 rescue 72 h cut.tif]

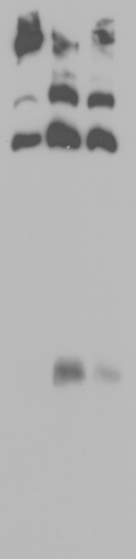

Supplement: Supplementary file 6 — Source data Fig. 2 [file 44319_2025_406_MOESM6_ESM.zip › Figure 2/Figure 2C/Rescue 72 h/Western of each antibody/UQCRC1 rescue 72 h cut.tif]

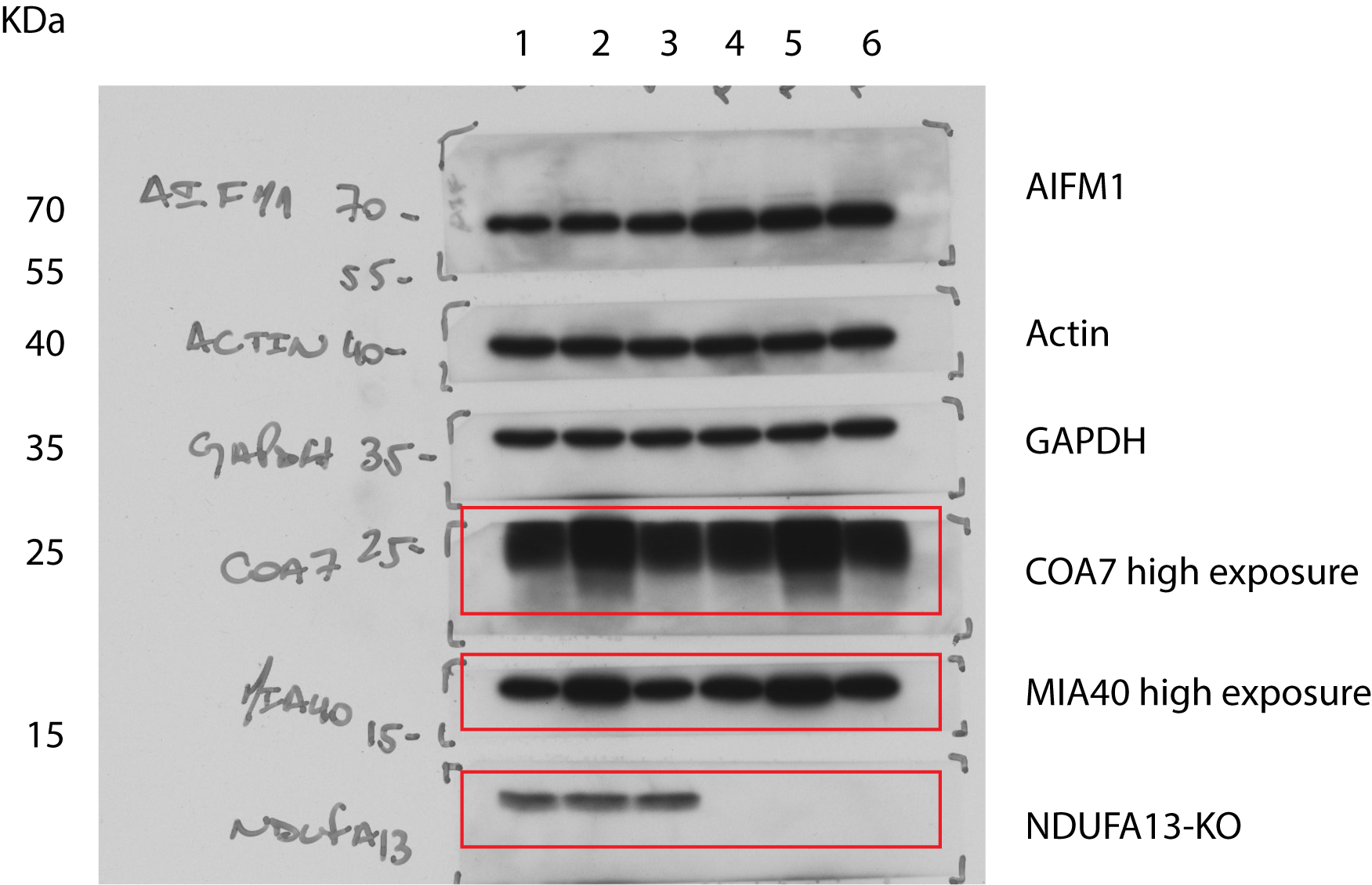

Supplement: Supplementary file 7 — Source data Fig. 3 [file 44319_2025_406_MOESM7_ESM.zip › Figure 3/Figure 3F/Original Western with cut area/MIA40 high exposure, COA7 high exposure and NDUFA13-KO original western.tif]

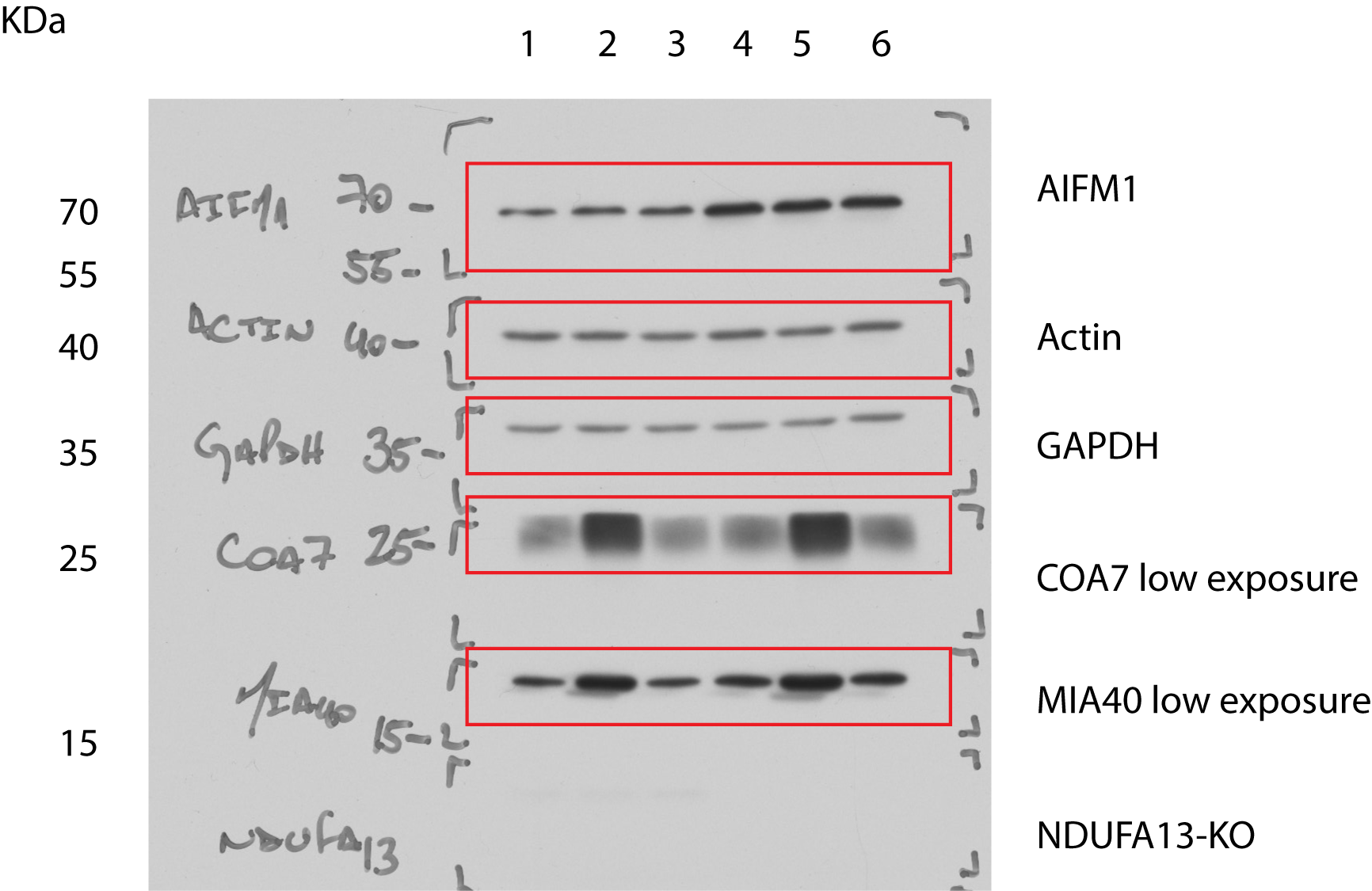

Supplement: Supplementary file 7 — Source data Fig. 3 [file 44319_2025_406_MOESM7_ESM.zip › Figure 3/Figure 3F/Original Western with cut area/MIA40 low exposure, COA7 low exposure, GAPDH, Actin and AIFM1 original western.tif]

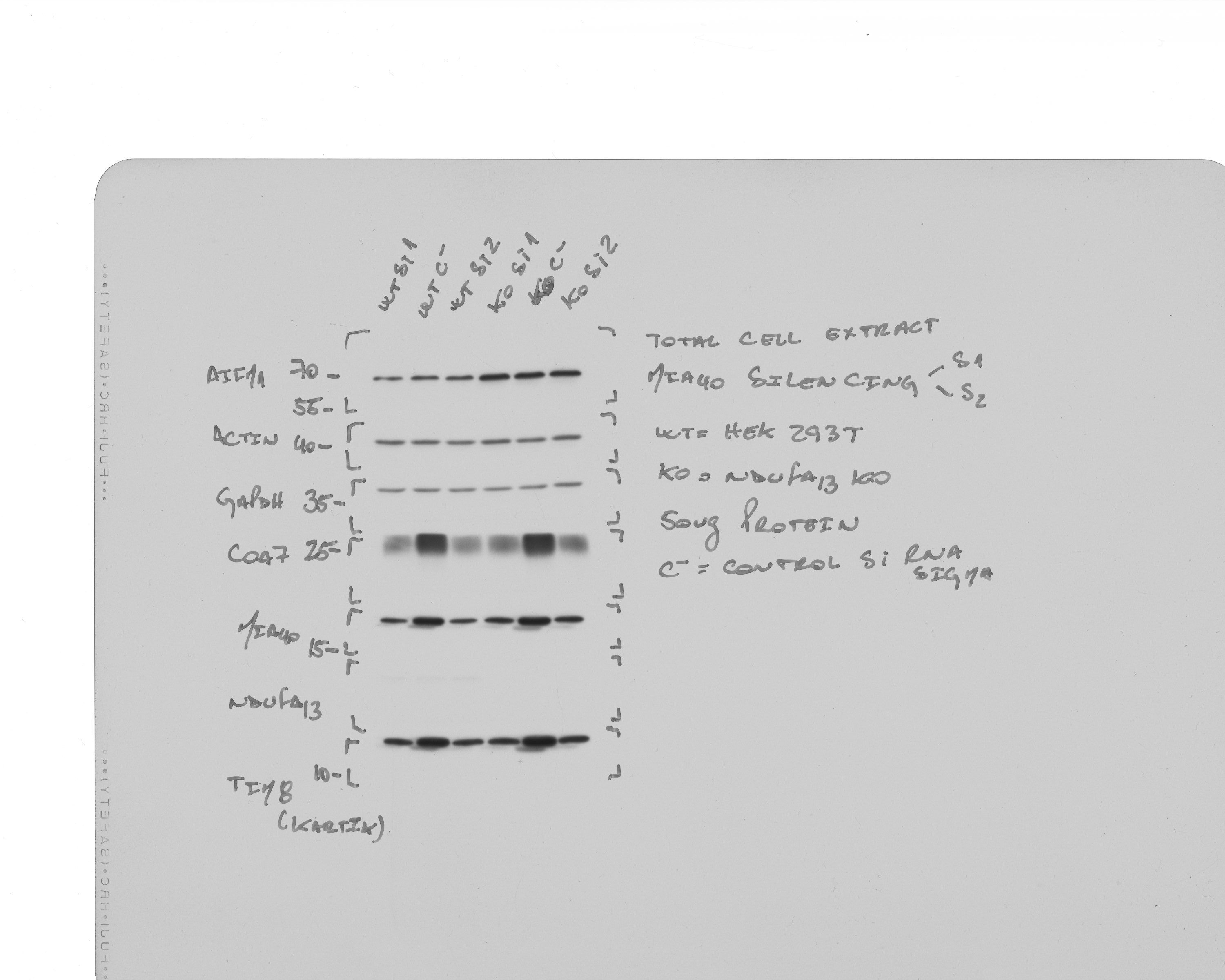

Supplement: Supplementary file 7 — Source data Fig. 3 [file 44319_2025_406_MOESM7_ESM.zip › Figure 3/Figure 3F/Original Western/siRNA018.jpg]

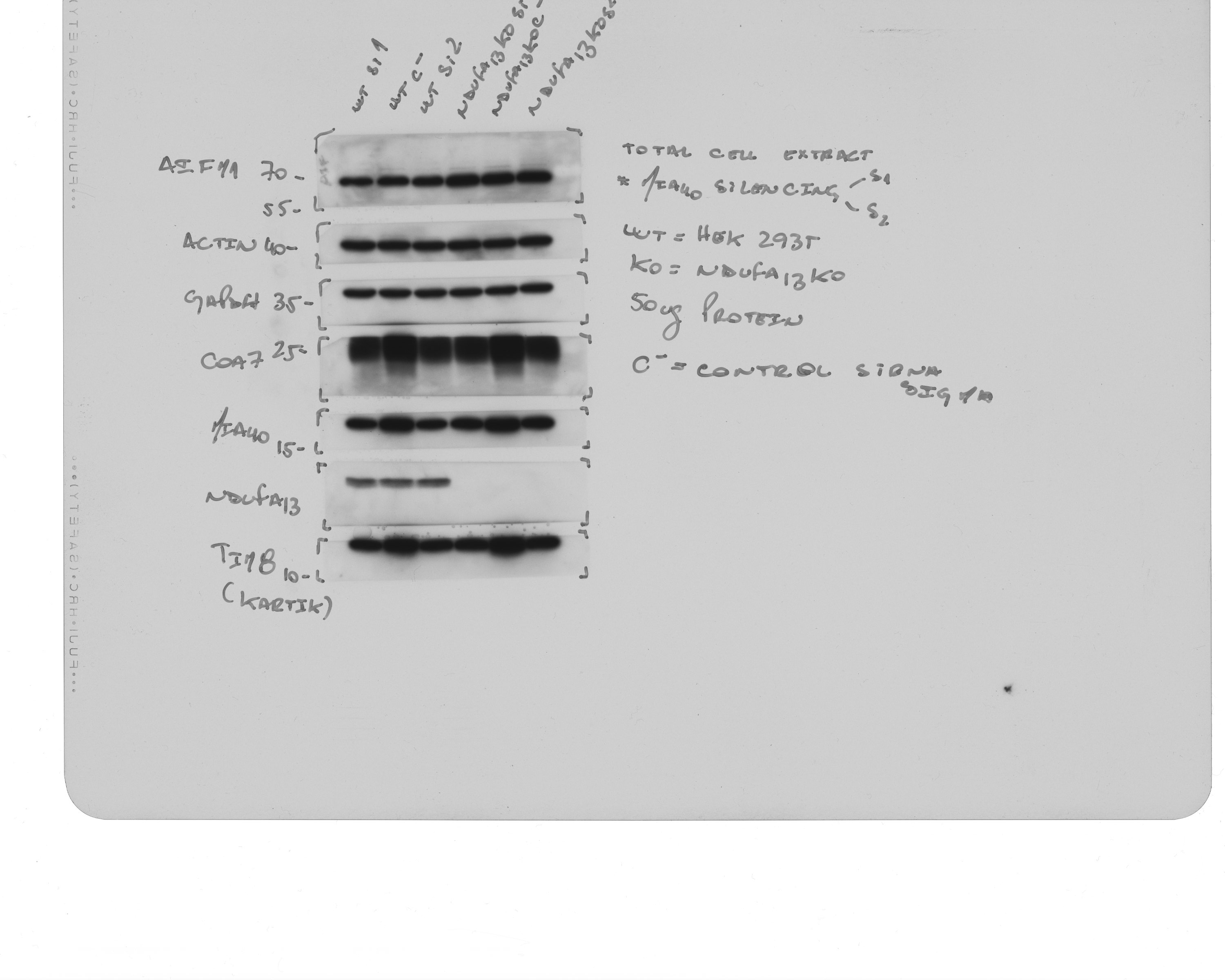

Supplement: Supplementary file 7 — Source data Fig. 3 [file 44319_2025_406_MOESM7_ESM.zip › Figure 3/Figure 3F/Original Western/siRNA022.jpg]

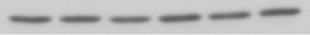

Supplement: Supplementary file 7 — Source data Fig. 3 [file 44319_2025_406_MOESM7_ESM.zip › Figure 3/Figure 3F/Western of cut for each antibody/Actin cut.tif]

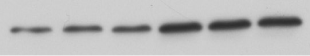

Supplement: Supplementary file 7 — Source data Fig. 3 [file 44319_2025_406_MOESM7_ESM.zip › Figure 3/Figure 3F/Western of cut for each antibody/AIFM1 cut.tif]

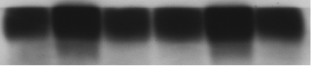

Supplement: Supplementary file 7 — Source data Fig. 3 [file 44319_2025_406_MOESM7_ESM.zip › Figure 3/Figure 3F/Western of cut for each antibody/COA7 high exposure cut.tif]

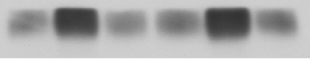

Supplement: Supplementary file 7 — Source data Fig. 3 [file 44319_2025_406_MOESM7_ESM.zip › Figure 3/Figure 3F/Western of cut for each antibody/COA7 low exposure cut.tif]

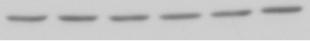

Supplement: Supplementary file 7 — Source data Fig. 3 [file 44319_2025_406_MOESM7_ESM.zip › Figure 3/Figure 3F/Western of cut for each antibody/GAPDH cut.tif]

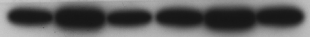

Supplement: Supplementary file 7 — Source data Fig. 3 [file 44319_2025_406_MOESM7_ESM.zip › Figure 3/Figure 3F/Western of cut for each antibody/MIA40 high exposure cut.tif]

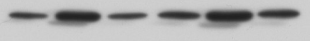

Supplement: Supplementary file 7 — Source data Fig. 3 [file 44319_2025_406_MOESM7_ESM.zip › Figure 3/Figure 3F/Western of cut for each antibody/MIA40 low exposure cut.tif]

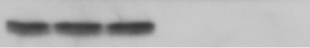

Supplement: Supplementary file 7 — Source data Fig. 3 [file 44319_2025_406_MOESM7_ESM.zip › Figure 3/Figure 3F/Western of cut for each antibody/NDUFA13 cut.tif]

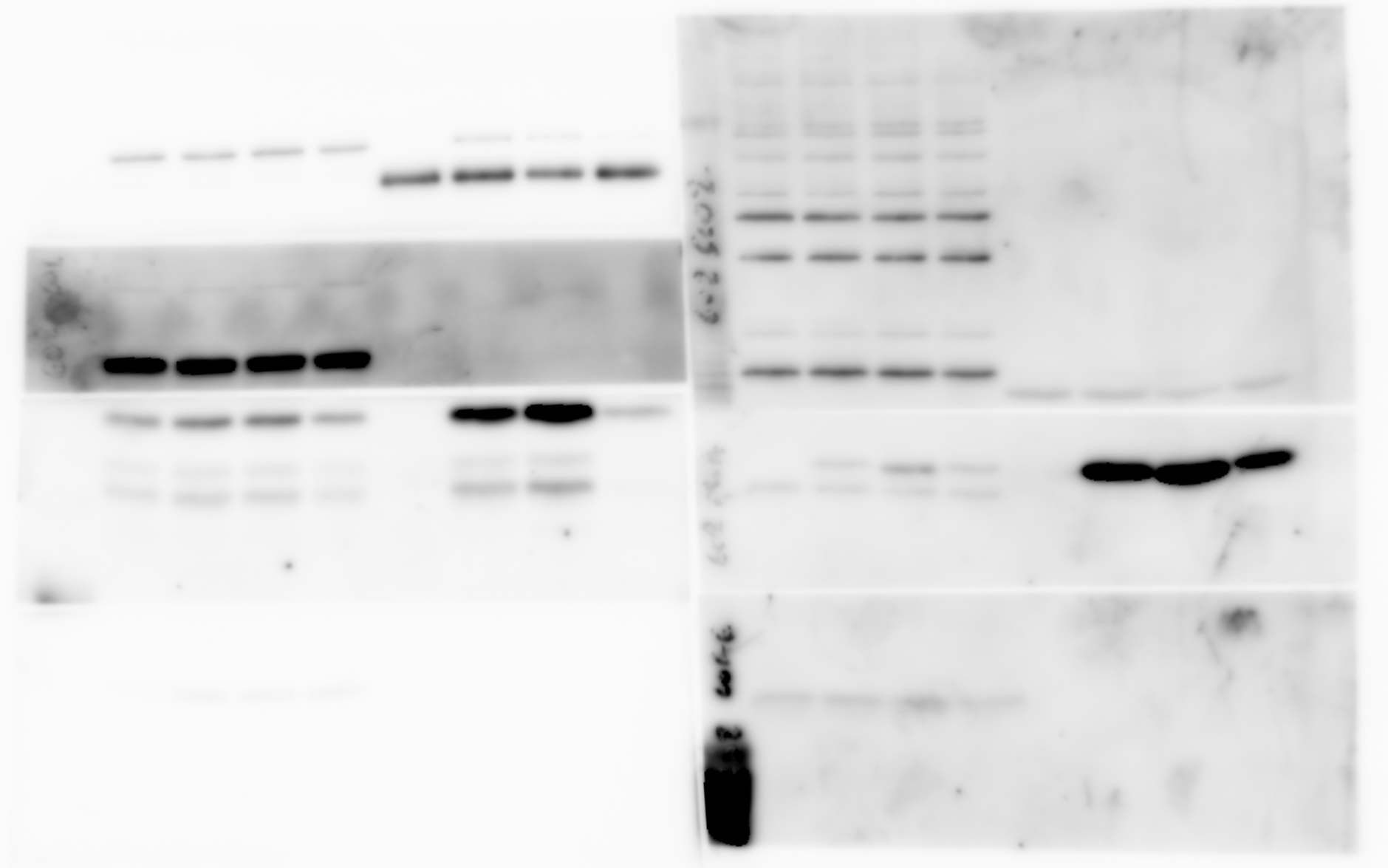

Supplement: Supplementary file 8 — Source data Fig. 4 [file 44319_2025_406_MOESM8_ESM.zip › Figure 4/Figure 4C/Figure 4C/Original Western with each cut area/MW608 2018.06.04_12.57.16-12_Ch_a.tif]

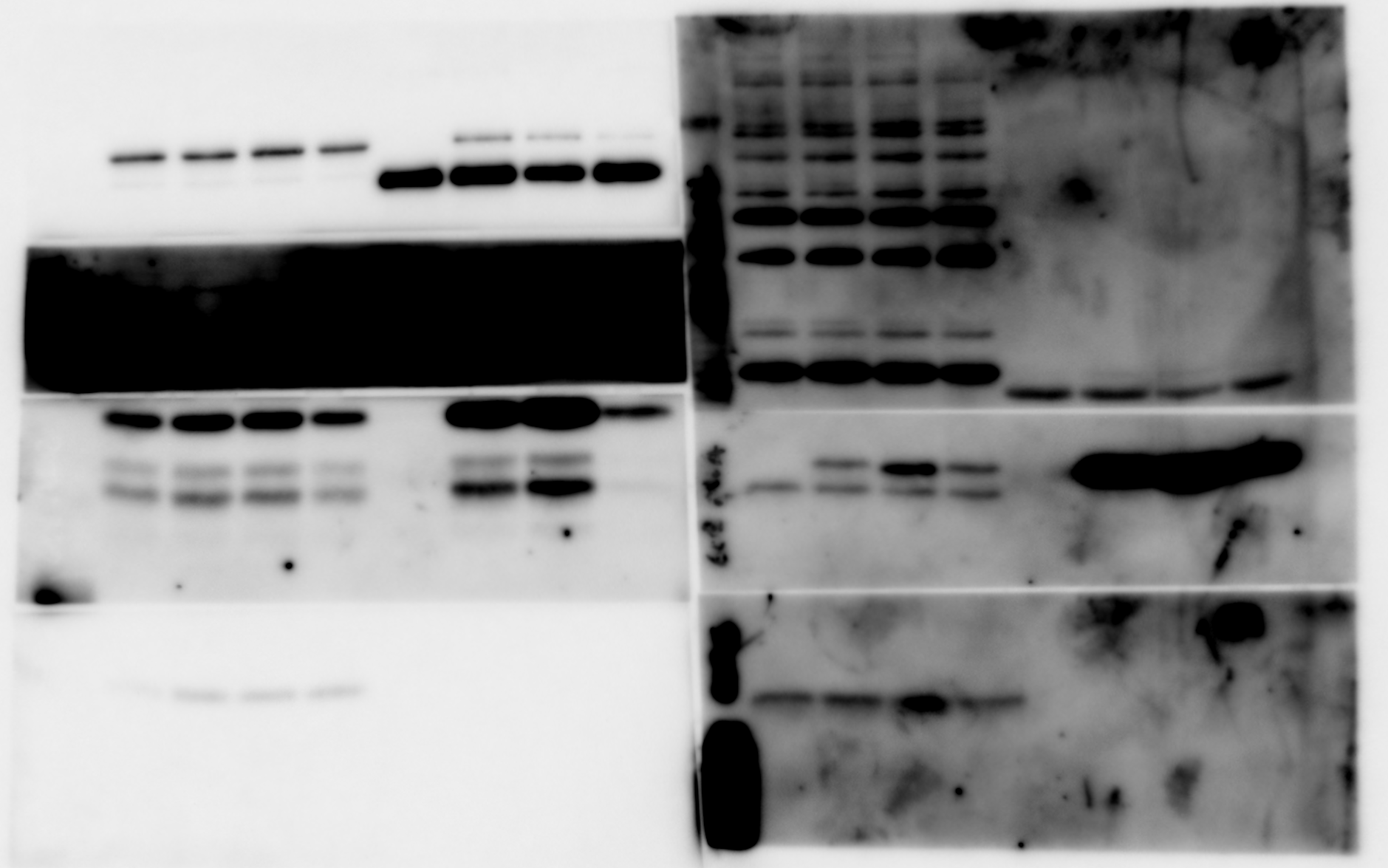

Supplement: Supplementary file 8 — Source data Fig. 4 [file 44319_2025_406_MOESM8_ESM.zip › Figure 4/Figure 4C/Figure 4C/Original Western with each cut area/MW608 2018.06.04_12.57.16-12_Ch_b.tif]

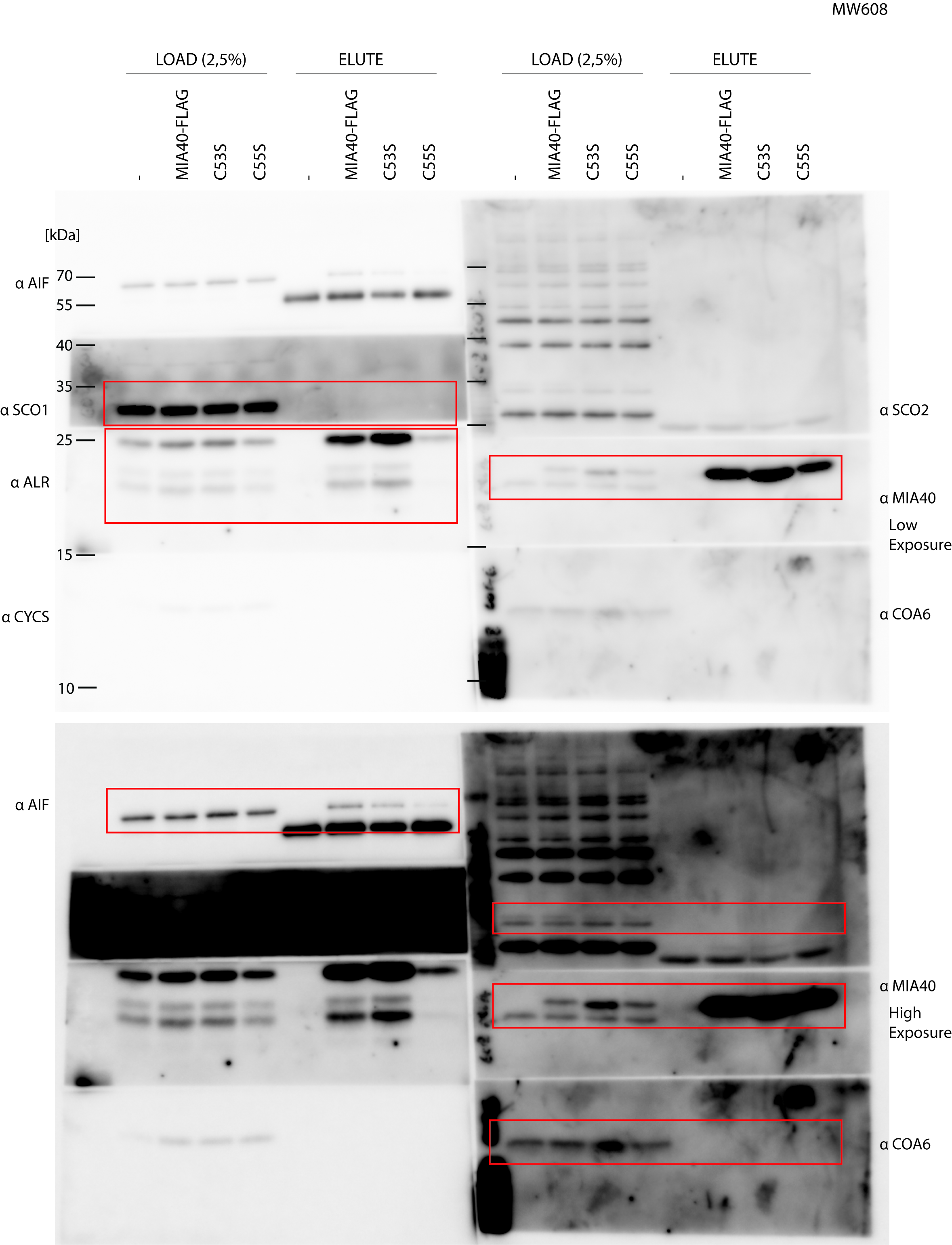

Supplement: Supplementary file 8 — Source data Fig. 4 [file 44319_2025_406_MOESM8_ESM.zip › Figure 4/Figure 4C/Figure 4C/Original Western/MIA40 high exposure, MIA40 low exposure, AIFM1, SCO1, SCO2, COA6, ALR.tif]

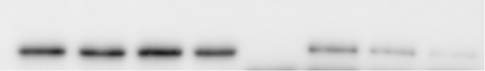

Supplement: Supplementary file 8 — Source data Fig. 4 [file 44319_2025_406_MOESM8_ESM.zip › Figure 4/Figure 4C/Figure 4C/Western of area cut for each antibody/AIFM1 cut.tif]

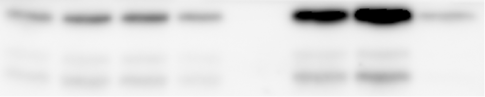

Supplement: Supplementary file 8 — Source data Fig. 4 [file 44319_2025_406_MOESM8_ESM.zip › Figure 4/Figure 4C/Figure 4C/Western of area cut for each antibody/ALR cut.tif]

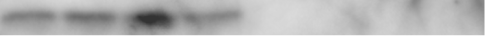

Supplement: Supplementary file 8 — Source data Fig. 4 [file 44319_2025_406_MOESM8_ESM.zip › Figure 4/Figure 4C/Figure 4C/Western of area cut for each antibody/COA6 cut.tif]

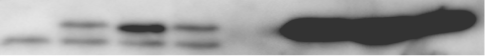

Supplement: Supplementary file 8 — Source data Fig. 4 [file 44319_2025_406_MOESM8_ESM.zip › Figure 4/Figure 4C/Figure 4C/Western of area cut for each antibody/MIA40 high exposure cut.tif]

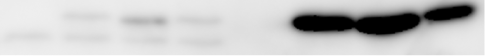

Supplement: Supplementary file 8 — Source data Fig. 4 [file 44319_2025_406_MOESM8_ESM.zip › Figure 4/Figure 4C/Figure 4C/Western of area cut for each antibody/MIA40 low exposure cut.tif]

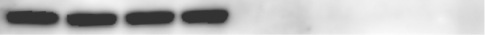

Supplement: Supplementary file 8 — Source data Fig. 4 [file 44319_2025_406_MOESM8_ESM.zip › Figure 4/Figure 4C/Figure 4C/Western of area cut for each antibody/SCO1 cut.tif]

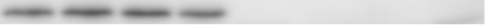

Supplement: Supplementary file 8 — Source data Fig. 4 [file 44319_2025_406_MOESM8_ESM.zip › Figure 4/Figure 4C/Figure 4C/Western of area cut for each antibody/SCO2 cut.tif]

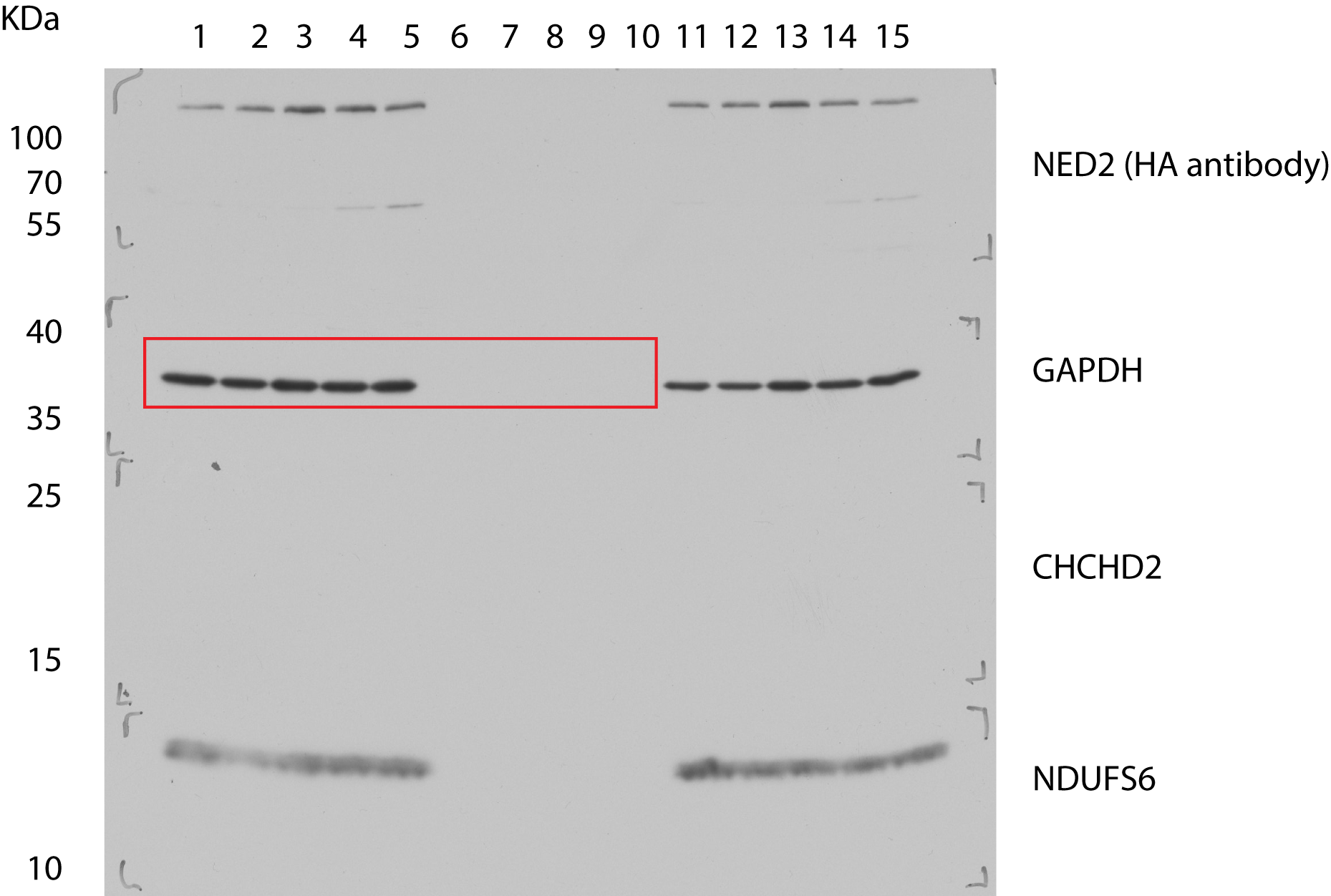

Supplement: Supplementary file 9 — Source data Fig. 5 [file 44319_2025_406_MOESM9_ESM.zip › Figure 5/Figure 5C/Original Western with each cut area/GAPDH original western.tif]

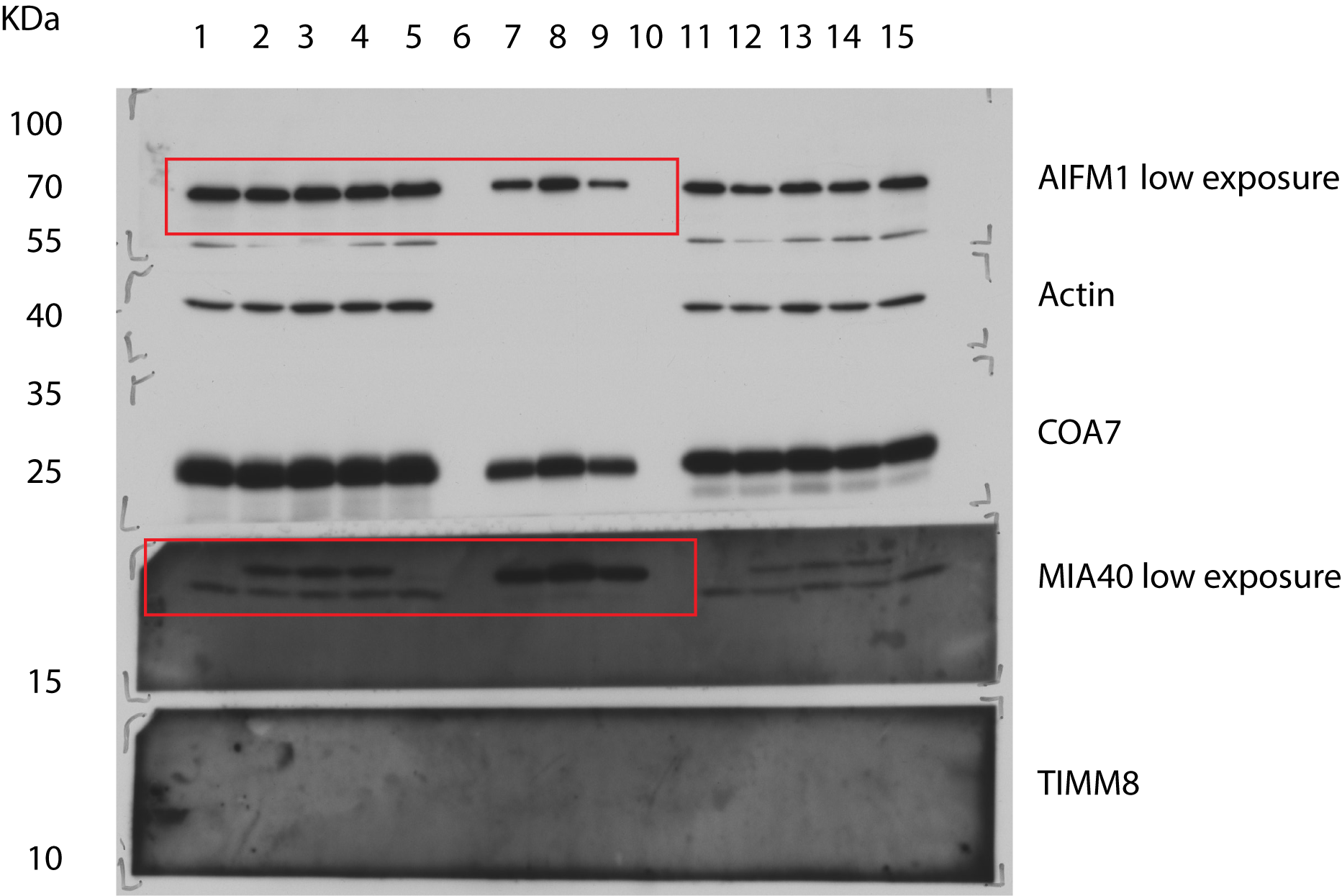

Supplement: Supplementary file 9 — Source data Fig. 5 [file 44319_2025_406_MOESM9_ESM.zip › Figure 5/Figure 5C/Original Western with each cut area/MIA40 high exposure exposure, AIFM1 high exposure original Western.tif]

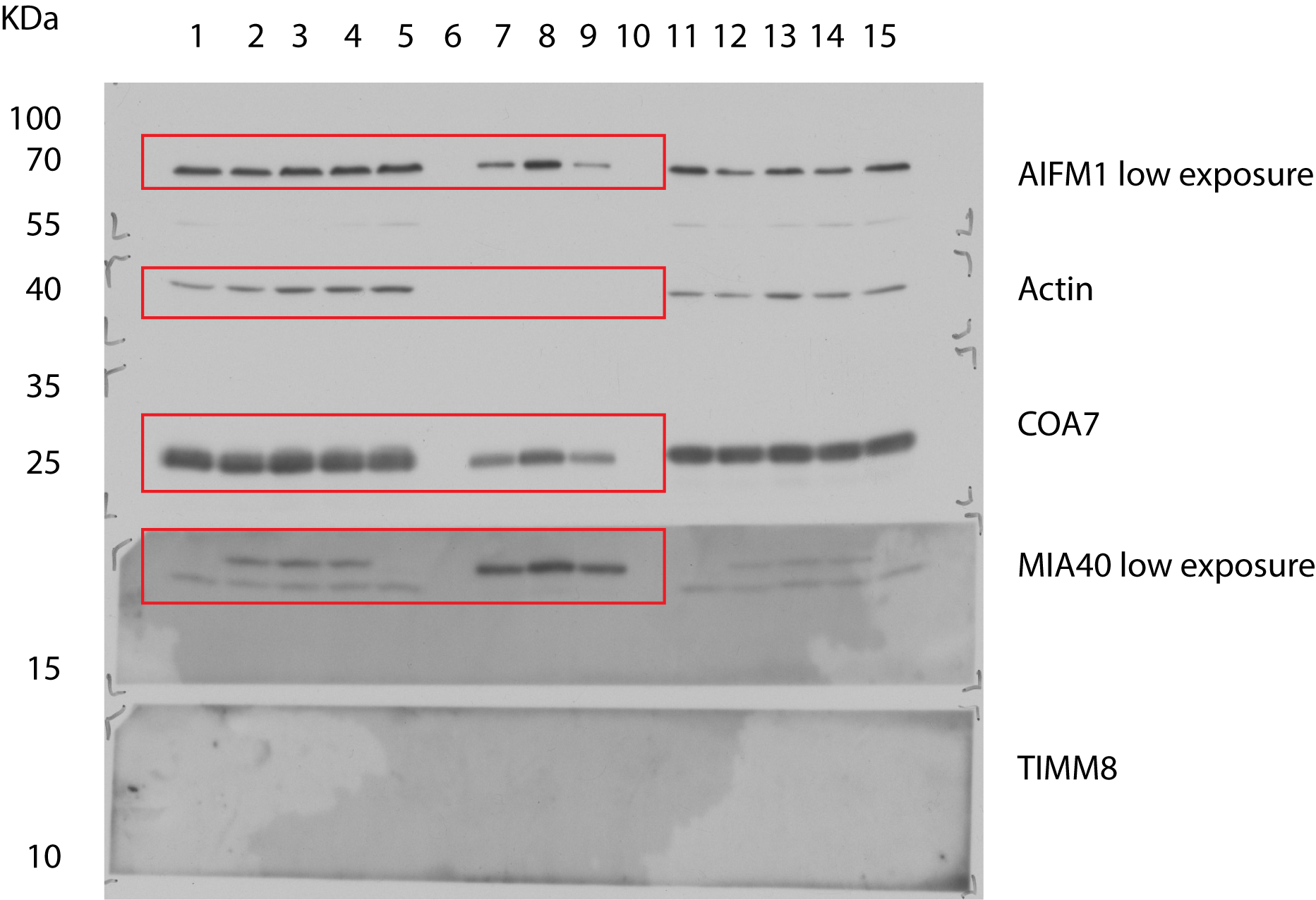

Supplement: Supplementary file 9 — Source data Fig. 5 [file 44319_2025_406_MOESM9_ESM.zip › Figure 5/Figure 5C/Original Western with each cut area/MIA40 low exposure, AIFM1 low exposure, COA7 and actin original Western.tif]

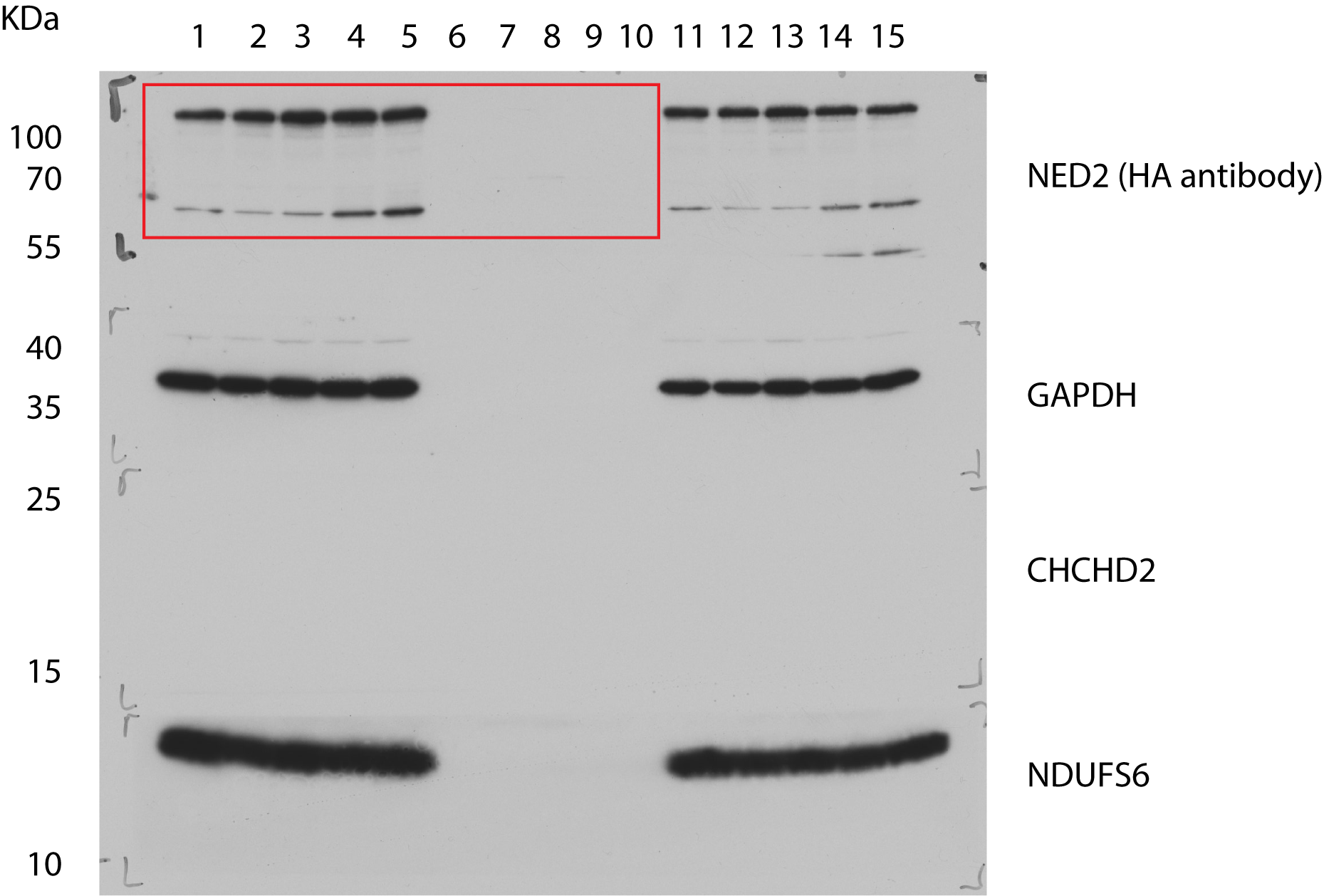

Supplement: Supplementary file 9 — Source data Fig. 5 [file 44319_2025_406_MOESM9_ESM.zip › Figure 5/Figure 5C/Original Western with each cut area/NED2-HA original western.tif]

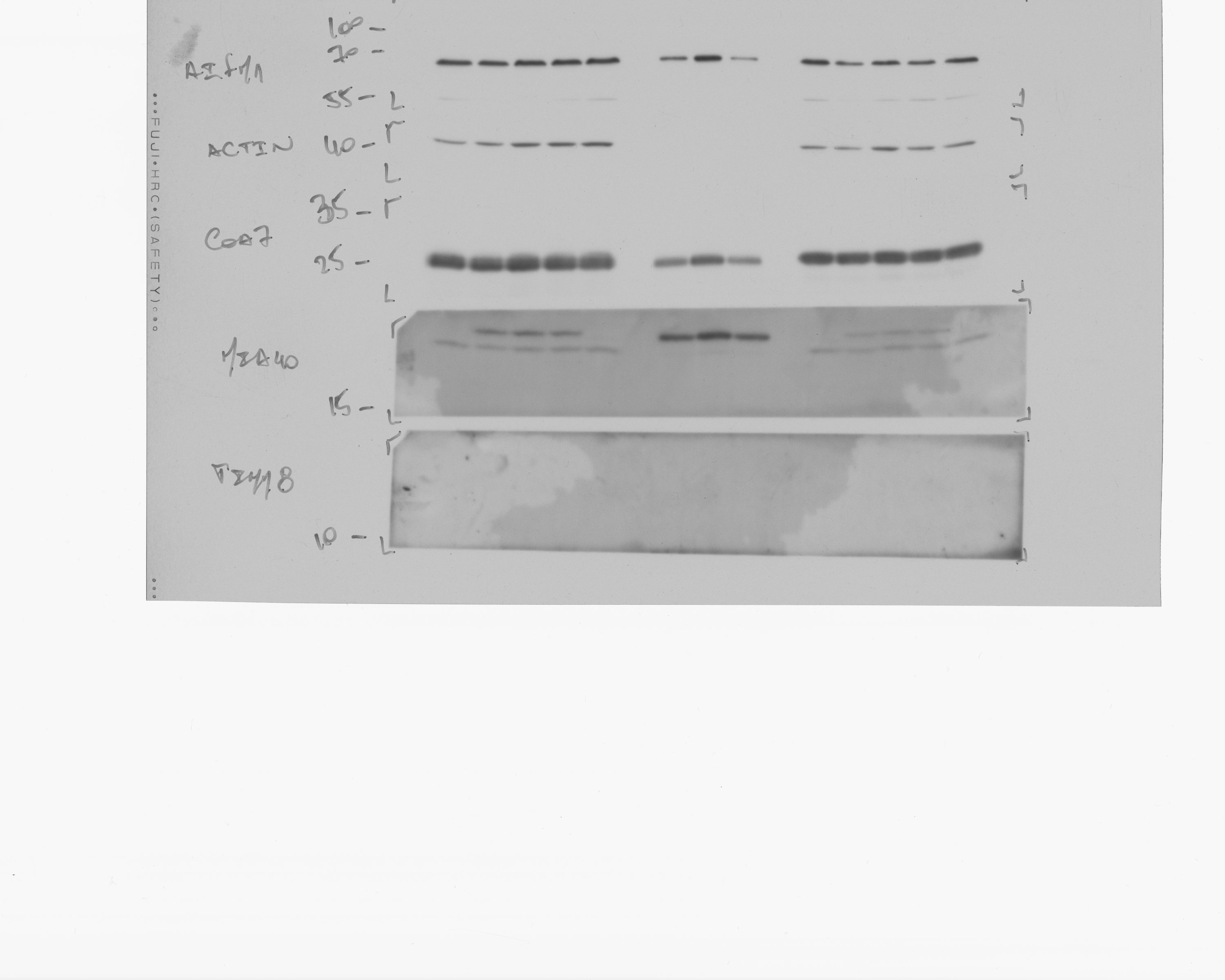

Supplement: Supplementary file 9 — Source data Fig. 5 [file 44319_2025_406_MOESM9_ESM.zip › Figure 5/Figure 5C/Original Western/NDE2 mia40033.jpg]

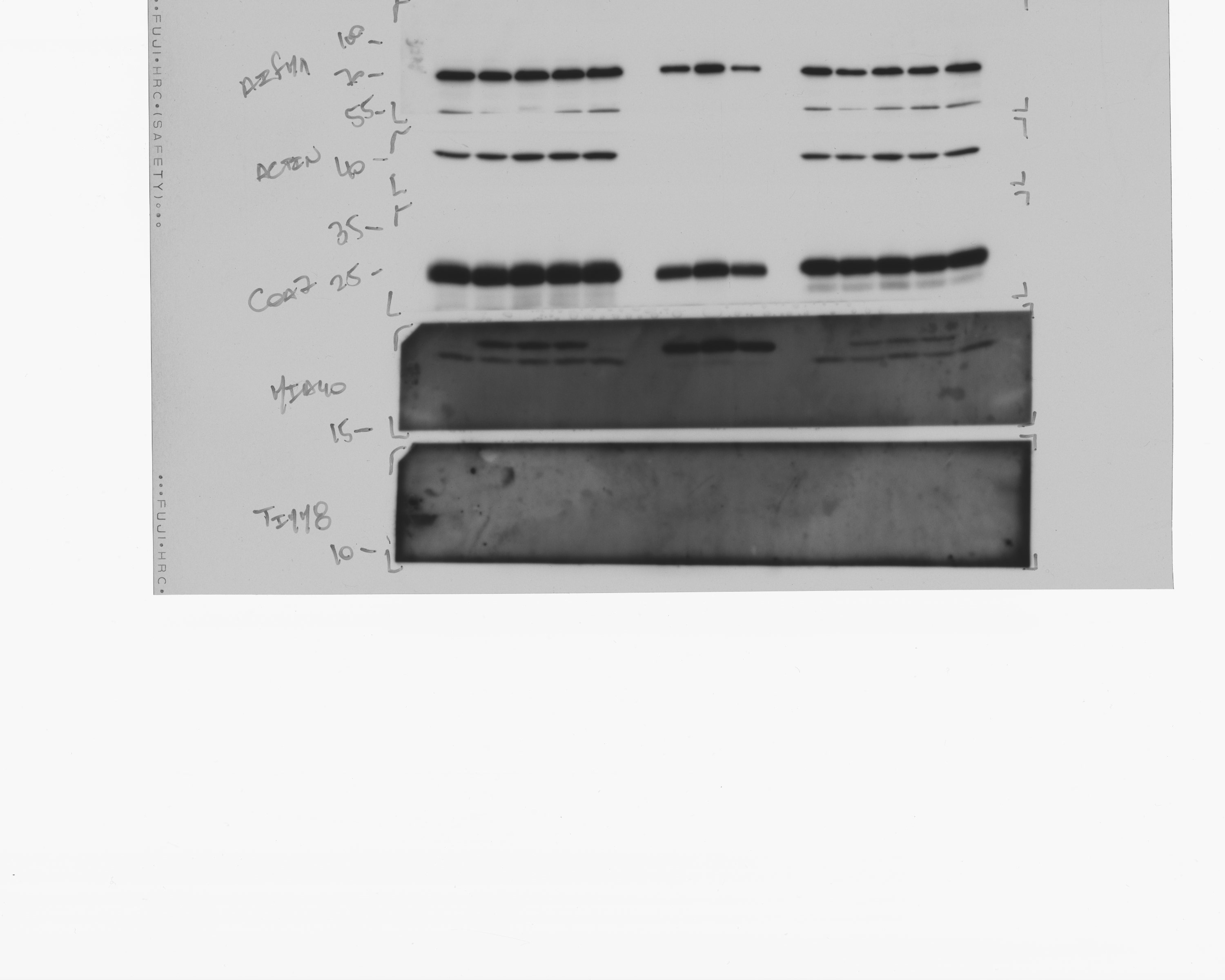

Supplement: Supplementary file 9 — Source data Fig. 5 [file 44319_2025_406_MOESM9_ESM.zip › Figure 5/Figure 5C/Original Western/NDE2 mia40034.jpg]

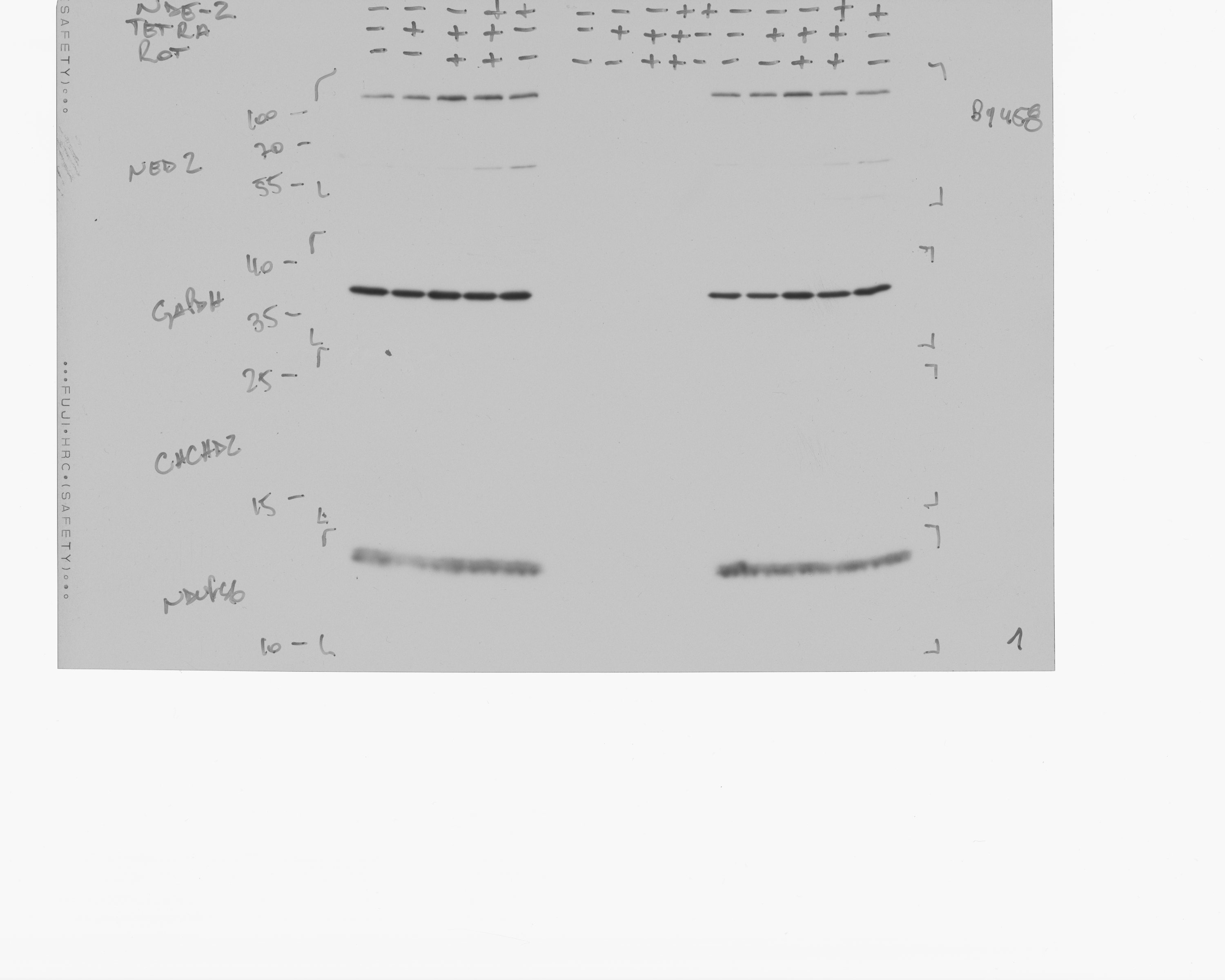

Supplement: Supplementary file 9 — Source data Fig. 5 [file 44319_2025_406_MOESM9_ESM.zip › Figure 5/Figure 5C/Original Western/NDE2026.jpg]

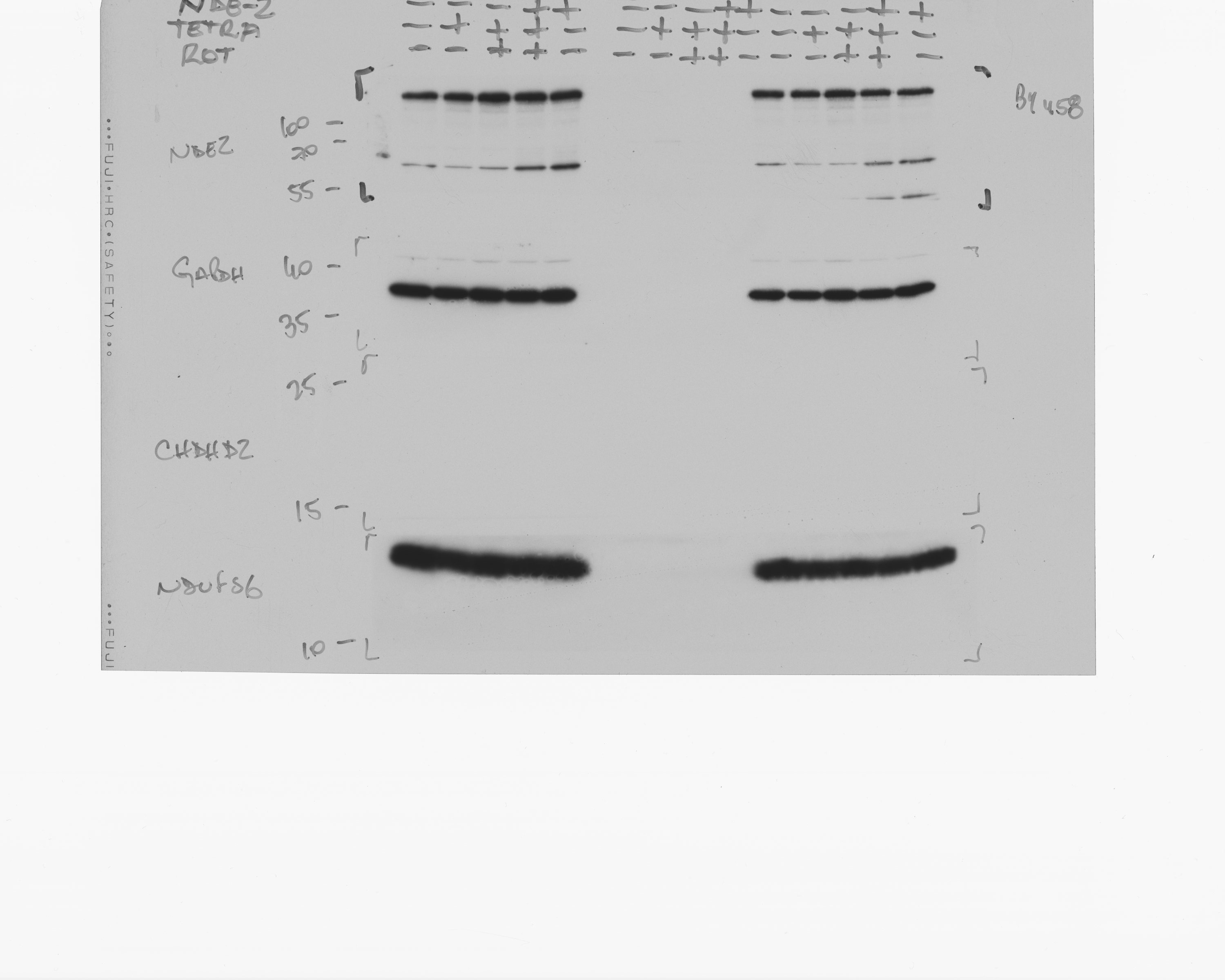

Supplement: Supplementary file 9 — Source data Fig. 5 [file 44319_2025_406_MOESM9_ESM.zip › Figure 5/Figure 5C/Original Western/NDE2028.jpg]

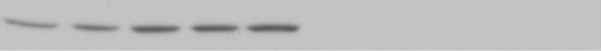

Supplement: Supplementary file 9 — Source data Fig. 5 [file 44319_2025_406_MOESM9_ESM.zip › Figure 5/Figure 5C/Western of area cut of each antibody/Actin cut.tif]

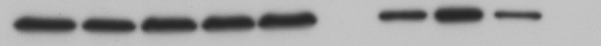

Supplement: Supplementary file 9 — Source data Fig. 5 [file 44319_2025_406_MOESM9_ESM.zip › Figure 5/Figure 5C/Western of area cut of each antibody/AIFM1 high exposure cut.tif]

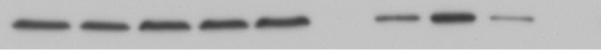

Supplement: Supplementary file 9 — Source data Fig. 5 [file 44319_2025_406_MOESM9_ESM.zip › Figure 5/Figure 5C/Western of area cut of each antibody/AIFM1 low exposure cut.tif]

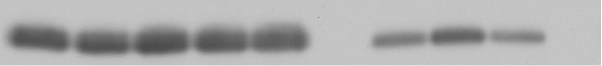

Supplement: Supplementary file 9 — Source data Fig. 5 [file 44319_2025_406_MOESM9_ESM.zip › Figure 5/Figure 5C/Western of area cut of each antibody/COA7.tif]

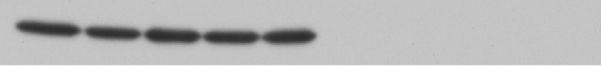

Supplement: Supplementary file 9 — Source data Fig. 5 [file 44319_2025_406_MOESM9_ESM.zip › Figure 5/Figure 5C/Western of area cut of each antibody/GAPDH cut.tif]

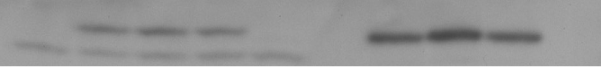

Supplement: Supplementary file 9 — Source data Fig. 5 [file 44319_2025_406_MOESM9_ESM.zip › Figure 5/Figure 5C/Western of area cut of each antibody/MIA40 high exposure cut.tif]

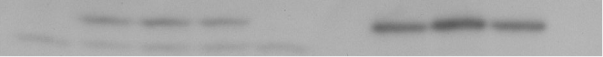

Supplement: Supplementary file 9 — Source data Fig. 5 [file 44319_2025_406_MOESM9_ESM.zip › Figure 5/Figure 5C/Western of area cut of each antibody/MIA40 low exposure cut.tif]

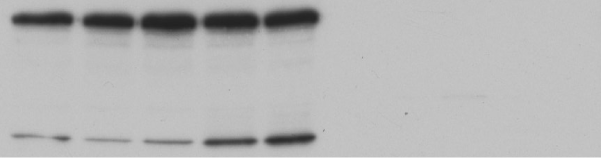

Supplement: Supplementary file 9 — Source data Fig. 5 [file 44319_2025_406_MOESM9_ESM.zip › Figure 5/Figure 5C/Western of area cut of each antibody/NED2-HA cut.tif]
