# Supplementary material for: MIA40 suppresses cell death induced by apoptosis-inducing factor 1
Source: EMBO Rep. 2025 Mar 7;26(7):1835–62. doi: 10.1038/s44319-025-00406-8 (PMC11976965; doi:10.1038/s44319-025-00406-8)
Supplement: Supplementary file 6 — Source data Fig. 2 [file 44319_2025_406_MOESM6_ESM.zip › Figure 2/Figure 2A/READ ME.docx]

FACS Accessory subunit KOs.

The file is labeled by the rescue followed by the name of the cell lines followed by transfection plasmid DNA followed by time of transfection and treatment.

Vehicle = DMSO 0.2%.

Cell death = cell death induced by Staurosporine in presence of caspase inhibitor.

FITC = Annexin V stained cells for gating.

PE = Propidium Iodide stained cells for gating.

FITC + PE = Annexin V + Propidium Iodide stained cells for gating.

Live negative unstained control = cells without FITC or PI for gating.

Live positive unstained control = cells boiled for 15 min at 65^o^C without FITC or PI for gating after.

Boiled positive control = cells boiled for 15 min at 65^o^C stained with respective markers.

pDNA = rescue with plasmid DNA to express NDUFA13 during 24 h or 72 h.

Empty vector = plasmid DNA without NDUFA13.
