# Supplementary material for: MIA40 suppresses cell death induced by apoptosis-inducing factor 1
Source: EMBO Rep. 2025 Mar 7;26(7):1835–62. doi: 10.1038/s44319-025-00406-8 (PMC11976965; doi:10.1038/s44319-025-00406-8)
Supplement: Supplementary file 6 — Source data Fig. 2 [file 44319_2025_406_MOESM6_ESM.zip › Figure 2/Figure 2C/Rescue 24 h/READ ME.docx]

READ ME

Line 1 = empty

Lines 2 to 4 = NDUFS1 (Complex I)

Lines 5 to 7 = UQCRC1 (Complex III)

Lines 8 to 10 = COXIV (complex IV)

2 = HEK293T transfected with empty vector during 24 h (WT)

3 = NDUFA-13KO transfected with empty vector during 24 h (Ko)

4 = NDUFA-13KO transfected with NDUFA13 during 24 h (R)

5 = HEK293T transfected with empty vector during 24 h (WT)

6 = NDUFA-13KO transfected with empty vector during 24 h (Ko)

7 = NDUFA-13KO transfected with NDUFA13 during 24 h (R)

8 = HEK293T transfected with empty vector during 24 h (WT)

9 = NDUFA-13KO transfected with empty vector during 24 h (Ko)

10 = NDUFA-13KO transfected with NDUFA13 during 24 h (R)
