# Supplementary material for: MIA40 suppresses cell death induced by apoptosis-inducing factor 1
Source: EMBO Rep. 2025 Mar 7;26(7):1835–62. doi: 10.1038/s44319-025-00406-8 (PMC11976965; doi:10.1038/s44319-025-00406-8)
Supplement: Supplementary file 6 — Source data Fig. 2 [file 44319_2025_406_MOESM6_ESM.zip › Figure 2/Figure 2C/Rescue 72 h/READ ME.docx]

READ ME

Lines 1 to 3 = COXIV (complex IV)

Lines 4 to 6 = ATP5B (ATP synthase)

Lines 7 to 9 = NDUFS1 (complex I)

Lines 10 to 12 = UQCRC1 (complex III)

Lines 13 to 16 = another experiment.

1 = HEK293T transfected with empty vector during 72 h (WT)

2 = NDUFA-13KO transfected with empty vector during 72 h (EV)

3 = NDUFA-13KO transfected with NDUFA13 during 72 h (NDUFA13)

4 = HEK293T transfected with empty vector during 72 h (WT)

5 = NDUFA-13KO transfected with empty vector during 72 h (EV)

6 = NDUFA-13KO transfected with NDUFA13 during 72 h (NDUFA13)

7 = HEK293T transfected with empty vector during 72 h (WT)

8 = NDUFA-13KO transfected with empty vector during 72 h (EV)

9 = NDUFA-13KO transfected with NDUFA13 during 72 h (NDUFA13)

10 = HEK293T transfected with empty vector during 72 h (WT)

11 = NDUFA-13KO transfected with empty vector during 72 h (EV)

12 = NDUFA-13KO transfected with NDUFA13 during 72 h (NDUFA13)
