# Supplementary material for: MIA40 suppresses cell death induced by apoptosis-inducing factor 1
Source: EMBO Rep. 2025 Mar 7;26(7):1835–62. doi: 10.1038/s44319-025-00406-8 (PMC11976965; doi:10.1038/s44319-025-00406-8)
Supplement: Supplementary file 7 — Source data Fig. 3 [file 44319_2025_406_MOESM7_ESM.zip › Figure 3/Figure 3F/READ ME.docx]

READ ME

Lines 1 = WTSi1 = HEK293T transfected with first oligonucleotide for MIA40 silencing described in material and methods table.

Line 2 = WT C- = HEK293T transfected with scramble universal control from sigma.

Line 3 = WTSi2 = HEK293T transfected with second oligonucleotide for MIA40 silencing described in material and methods table.

Lines 4 = KOSi1 = NDUFA13-KO transfected with first oligonucleotide for MIA40 silencing described in material and methods table.

Line 5 = KO C- = NDUFA13-KO transfected with scramble universal control from sigma.

Line 6 = KOSi2 = NDUFA13-KO transfected with second oligonucleotide for MIA40 silencing described in material and methods table.
