# Supplementary material for: MIA40 suppresses cell death induced by apoptosis-inducing factor 1
Source: EMBO Rep. 2025 Mar 7;26(7):1835–62. doi: 10.1038/s44319-025-00406-8 (PMC11976965; doi:10.1038/s44319-025-00406-8)
Supplement: Supplementary file 8 — Source data Fig. 4 [file 44319_2025_406_MOESM8_ESM.zip › Figure 4/Figure 4A/READ ME.docx]

FACS Accessory subunit KOs.

The file is labeled by the AIFM1 inhibitors followed by cell line followed by treatment.

Vehicle = DMSO 0.2%.

Cell death = cell death induced by Staurosporine in presence of caspase inhibitor.

MIA40WT = Cells expressing MIA40Flag

MIA40C53S = Cells expressing MIA40C53SFlag variant

MIA40C55S = Cells expressing MIA40C55SFlag variant

Flp-inTM T-REXtm 293 = Cells expressing only native MIA40

FITC = Annexin V stained cells for gating.

PE = Propidium Iodide stained cells for gating.

FITC + PE = Annexin V + Propidium Iodide stained cells for gating.

Live negative unstained control = cells without FITC or PI for gating.

Live positive unstained control = cells boiled for 15 min at 65^o^C without FITC or PI for gating after.

Boiled positive control = cells boiled for 15 min at 65^o^C stained with respective markers.
