# Supplementary material for: MIA40 suppresses cell death induced by apoptosis-inducing factor 1
Source: EMBO Rep. 2025 Mar 7;26(7):1835–62. doi: 10.1038/s44319-025-00406-8 (PMC11976965; doi:10.1038/s44319-025-00406-8)
Supplement: Supplementary file 8 — Source data Fig. 4 [file 44319_2025_406_MOESM8_ESM.zip › Figure 4/Figure 4C/Figure 4C/READ ME.docx]

READ ME

- = No MIA40_FLAg_ induction

MIA40FLAG = Induction of MIA40_FLAg_

C53S = Induction of MIA40C53S_FLAg_ variant

C55S = Induction of MIA40C55S_FLAg_ variant
