# Supplementary material for: MIA40 suppresses cell death induced by apoptosis-inducing factor 1
Source: EMBO Rep. 2025 Mar 7;26(7):1835–62. doi: 10.1038/s44319-025-00406-8 (PMC11976965; doi:10.1038/s44319-025-00406-8)
Supplement: Supplementary file 9 — Source data Fig. 5 [file 44319_2025_406_MOESM9_ESM.zip › Figure 5/Figure 5A/READ ME.docx]

FACS Accessory subunit KOs.

The file is labeled by NED2-HA followed by cell line followed by transfection.

Vehicle = DMSO 0.2%.

Cell death = cell death induced by Staurosporine in presence of caspase inhibitor.

FITC = Annexin V stained cells for gating.

PE = Propidium Iodide stained cells for gating.

FITC + PE = Annexin V + Propidium Iodide stained cells for gating.

Live negative unstained control = cells without FITC or PI for gating.

Live positive unstained control = cells boiled for 15 min at 65^o^C without FITC or PI for gating after.

Boiled positive control = cells boiled for 15 min at 65^o^C stained with respective markers.

NED2pDNA = Cells transfected with NED2 plasmid DNA.

Empty vector = Cells transfected with empty vector.
