# Supplementary material for: MIA40 suppresses cell death induced by apoptosis-inducing factor 1
Source: EMBO Rep. 2025 Mar 7;26(7):1835–62. doi: 10.1038/s44319-025-00406-8 (PMC11976965; doi:10.1038/s44319-025-00406-8)
Supplement: Supplementary file 9 — Source data Fig. 5 [file 44319_2025_406_MOESM9_ESM.zip › Figure 5/Figure 5C/READ ME.docx]

READ ME

Lines 1 to 5 = Load

Lines 6 to 10 = Eluate

Lines 11 to 15 = Unbound

Line 1 = No induction of MIA40, no rotenone treatment, no NED2-HA transfection.

Line 2 = Induction of MIA40, no rotenone treatment, no NED2-HA transfection.

Line 3 = Induction of MIA40, rotenone treatment, no NED2-HA transfection.

Line 4 = Induction of MIA40, rotenone treatment, NED2-HA transfection.

Line 5 = No Induction of MIA40, rotenone treatment, NED2-HA transfection.

Line 6 = No induction of MIA40, no rotenone treatment, no NED2-HA transfection.

Line 7 = Induction of MIA40, no rotenone treatment, no NED2-HA transfection.

Line 8 = Induction of MIA40, rotenone treatment, no NED2-HA transfection.

Line 9 = Induction of MIA40, rotenone treatment, NED2-HA transfection.

Line 10 = No Induction of MIA40, rotenone treatment, NED2-HA transfection.

Line 11 = No induction of MIA40, no rotenone treatment, no NED2-HA transfection.

Line 12 = Induction of MIA40, no rotenone treatment, no NED2-HA transfection.

Line 13 = Induction of MIA40, rotenone treatment, no NED2-HA transfection.

Line 14 = Induction of MIA40, rotenone treatment, NED2-HA transfection.

Line 15 = No Induction of MIA40, rotenone treatment, NED2-HA transfection.
