# Supplementary material for: MIA40 suppresses cell death induced by apoptosis-inducing factor 1
Source: EMBO Rep. 2025 Mar 7;26(7):1835–62. doi: 10.1038/s44319-025-00406-8 (PMC11976965; doi:10.1038/s44319-025-00406-8)
Supplement: Supplementary file 10 — Expanded View Figures [file 44319_2025_406_MOESM10_ESM.pdf]

Expanded View Figures

Figure EV1. Complex I accessory subunits KO cells screen for metabolic profile and resistance to AIFM1-induced cell death.

(**A**) control HEK293T cells and correspondent complex I accessory subunits knock outs (KO) cell lines viability accessed by The CellTiter 96® AQueous One Solution Reagent after cell death induced by AIFM1. Data shown are mean ± SEM ($n = 3$ biological replicates) Statistical significance was obtained by an ordinary one-way ANOVA with Bonferroni multiple comparisons test (compared to HEK293T: NDUFA7-KO, $p > 0.9999$; NDUFS6-KO, $p > 0.9999$; NDUFV3-KO, $p = 0.0003$; NDUFA13-KO, $p = 0.0012$; NDUFA5-KO, $p = 0.0153$; NDUFS5-KO, $p > 0.9999$; NDUFB4-KO, $p = 0.0064$; NDUFB7-KO, $p > 0.9999$). *$p < 0.05$ indicates a significant difference compared to the HEK293T cells. (**B**) control HEK293T cells and correspondent complex I accessory subunits KO cell lines survival accessed by sulforhodamine b after cell death induced by AIFM1. Data shown are mean ± SEM ($n = 3$ biological replicates). Statistical significance was obtained by an ordinary one-way ANOVA with Bonferroni multiple comparisons test (compared to HEK293T: NDUFA7-KO, $p > 0.9999$; NDUFS6-KO, $p > 0.9999$; NDUFV3-KO, $p = 0.0002$; NDUFA13-KO, $p = 0.0002$; NDUFA5-KO, $p = 0.0489$; NDUFS5-KO, $p > 0.9999$; NDUFB4-KO, $p = 0.0314$; NDUFB7-KO, $p > 0.9999$). *$p < 0.05$ indicates a significant difference compared to the HEK293T cells. (**C**) Complex I activity of the control HEK293T cells and correspondent complex I accessory subunits KO cell lines. Data shown are mean ± SEM ($n = 3$ biological replicates). Statistical significance was obtained by an ordinary one-way ANOVA with Bonferroni multiple comparisons test (compared to HEK293T: NDUFA7-KO, $p < 0.0001$; NDUFS6-KO, $p < 0.0001$; NDUFV3-KO, $p < 0.0001$; NDUFA13-KO, $p < 0.0001$; NDUFA5-KO, $p < 0.0001$; NDUFS5-KO, $p < 0.0001$; NDUFB4-KO, $p < 0.0001$; NDUFB7-KO, $p < 0.0001$). *$p < 0.05$ indicates a significant difference compared to the HEK293T cells. (**D**) NADH/NAD^+ balance of the control HEK293T cells and correspondent complex I accessory subunits KO cell lines. Data shown are mean ± SEM ($n = 3$ biological replicates). Statistical significance was obtained by an ordinary one-way ANOVA with Bonferroni multiple comparisons test (compared to HEK293T: NDUFA7-KO, $p = 0.5571$; NDUFS6-KO, $p = 0.0476$; NDUFV3-KO, $p = 0.0007$; NDUFA13-KO, $p = 0.0018$; NDUFA5-KO, $p = 0.9997$; NDUFS5-KO, $p = 0.7218$; NDUFB4-KO, $p = 0.8921$; NDUFB7-KO, $p = 0.6560$). *$p < 0.05$ indicates a significant difference compared to the HEK293T cells. (**E**) control HEK293T, NDUFA13- and NDUFB4-KO cell lines metabolic consumption of 31 different metabolites. Data shown are mean ± SEM ($n = 4$ or 3 biological replicates per substrate). Statistical significance was obtained by an ordinary two-way ANOVA with Bonferroni multiple comparisons test. For succinate oxidation, $p < 0.0001$. For fumaric acid oxidation, $p = 0.0207$. For pyruvic acid + L-malic acid 100 µM oxidation, $p = 0.0390$. # $p < 0.05$ indicates difference between NDUFA13-KO and NDFUB4-KO. The remaining statistical comparisons are presented in Table EV3. (**F**) Cellular protein extracts were isolated from the control HEK293T cells and correspondent complex I accessory subunits KO cell lines. The samples were analyzed by reducing SDS–PAGE and Western blot. (**G**) Quantification of AIFM1 expression from (**F**) using ImageJ. Data shown are mean ± SEM ($n = 3$ biological replicates). Statistical significance was obtained by an ordinary one-way ANOVA with Bonferroni multiple comparisons test (compared to HEK293T: NDUFV3-KO, $p = 0.0015$; NDUFA5-KO, $p > 0.9999$; NDUFA13-KO; $p < 0.0001$; NDUFB4-KO; $p > 0.9999$). *$p < 0.001$ indicates a significant difference compared to the HEK293T cells. (**H**) Localization of mitochondrial proteins analyzed by limited degradation by proteinase K in intact mitochondria (250 mM sucrose) or mitoplasts (100, 25, and 5 mM sucrose). The samples were analyzed by SDS–PAGE and Western blot. OM, outer membrane; IM, inner membrane; IMS, intermembrane space.

© The Author(s)

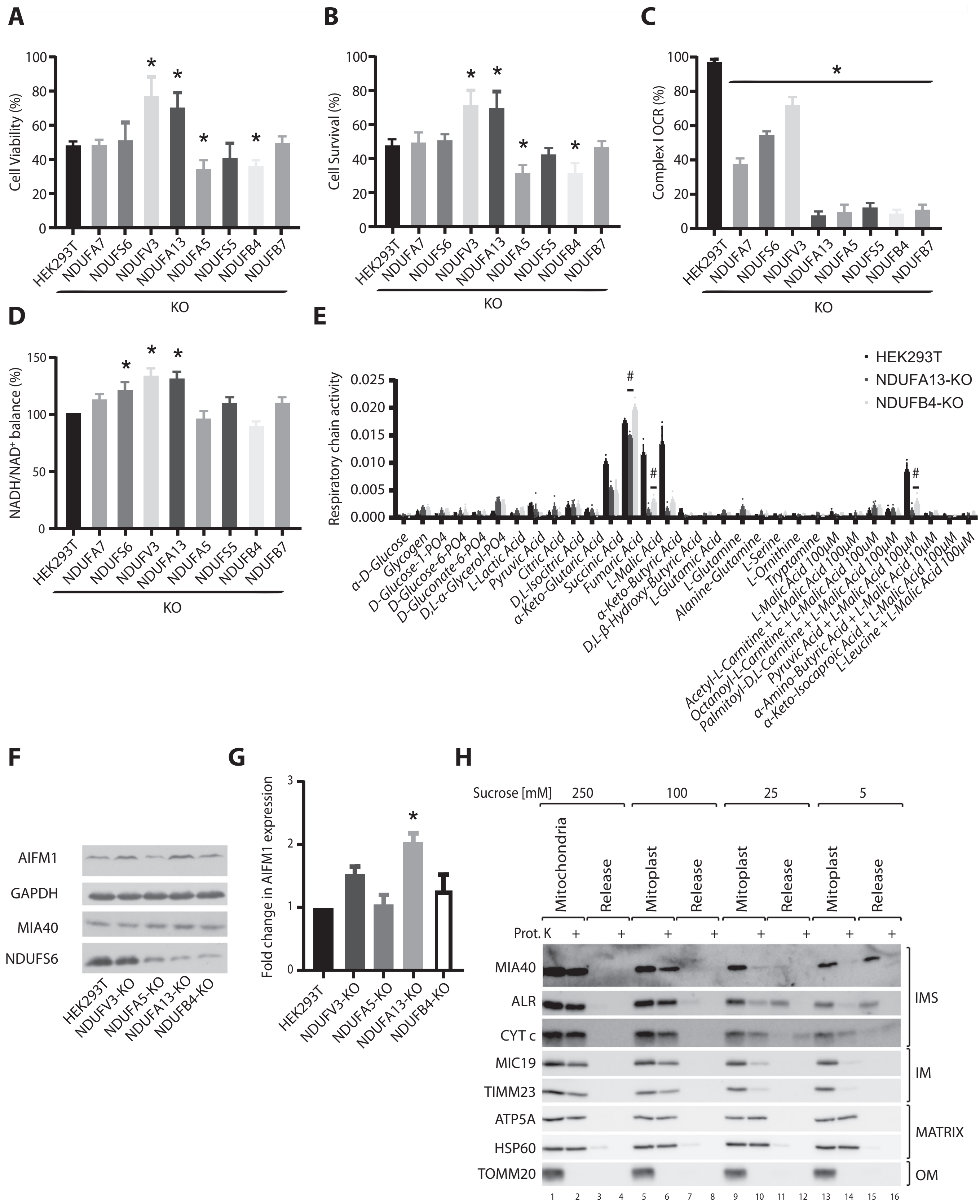
A
Cell Viability (%)
HEK293T
NDUFA7
NDUFS6
NDUFV3
NDUFA13
NDUFA5
NDUFS5
NDUFB4
NDUFB7
KO
B
Cell Survival (%)
C
Complex I OCR (%)
D
NADH/NAD+ balance (%)
E
Respiratory chain activity
HEK293T
NDUFA13-KO
NDUFB4-KO
α-D-Glucose
Glycogen
D-Glucose-1-PO4
D-Glucose-6-PO4
D-Gluconate-6-PO4
D,L-α-Glycerol-PO4
L-Lactic Acid
Pyruvic Acid
Citric Acid
D,L-Isocitric Acid
α-Keto-Glutaric Acid
Succinic Acid
Fumaric Acid
L-Malic Acid
α-Keto-Butyric Acid
D,L-β-Hydroxy-Butyric Acid
L-Glutamic Acid
L-Glutamine
Alanine-Glutamine
L-Serine
L-Ornithine
Tryptamine
L-Malic Acid 100µM
Acetyl-L-Carnitine + L-Malic Acid 100µM
Octanoyl-L-Carnitine + L-Malic Acid 100µM
Palmitoyl-D,L-Carnitine + L-Malic Acid 100µM
Pyruvic Acid + L-Malic Acid 100µM
α-Amino-Butyric Acid + L-Malic Acid 10µM
α-Keto-Isocaproic Acid + L-Malic Acid 100µM
L-Leucine + L-Malic Acid 100µM
F
AIFM1
GAPDH
MIA40
NDUFS6
HEK293T
NDUFV3-KO
NDUFA5-KO
NDUFA13-KO
NDUFB4-KO
G
Fold change in AIFM1 expression
H
Sucrose [mM] 250 100 25 5
Mitochondria
Release
Mitoplast
Prot. K
MIA40
ALR
CYT c
MIC19
TIMM23
ATP5A
HSP60
TOMM20
IMS
IM
MATRIX
OM
1 2 3 4 5 6 7 8 9 10 11 12 13 14 15 16

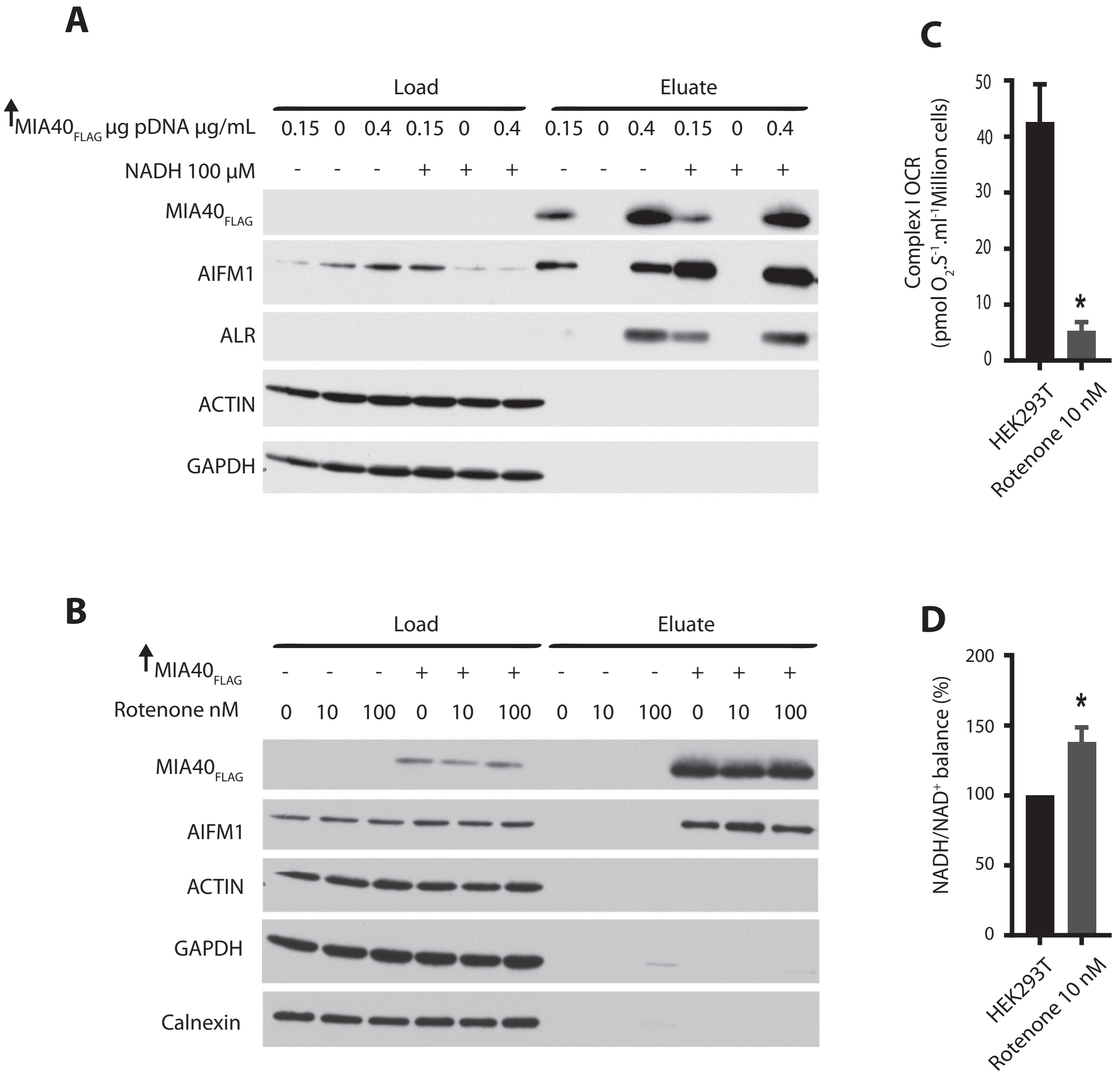

Figure EV2. NADH increases AIFM1 and MIA40 interaction.

(**A**) control HEK293T cells transfected with an empty plasmid or MIA40$_{FLAG}$ were solubilized, and the affinity purification of MIA40$_{FLAG}$ was performed in the presence of 100 µM NADH. Fractions were analyzed by SDS-PAGE and Western blot. Load 2.5%; Eluate: 100%. (**B**) HEK293T cells were transfected with an empty plasmid or MIA40$_{FLAG}$ for 48 h and then were incubated with 10 nM or 100 nM rotenone for 12 h. Afterwards, the cells were solubilized and the affinity purification of MIA40$_{FLAG}$ was performed. Fractions were analyzed by SDS–PAGE and Western blot. Load 2.5%; Eluate: 100%. (**C**) HEK293T cells complex I activity after 12 h treatment of 10 nM rotenone. Data shown are mean ± SEM ($n = 3$ biological replicates). Statistical significance was obtained by two-tailed unpaired t-test $p = 0.0007$. *$p < 0.05$ indicates significant differences between groups. (**D**) HEK293T cells were treated with 10 nM rotenone for 12 h and afterwards, the NADH/NAD$^+$ balance was measured. Data shown are mean ± SEM ($n = 3$ biological replicates). Statistical significance was obtained by two-tailed unpaired t-test $p = 0.0034$. *$p < 0.05$ indicates significant differences between groups.

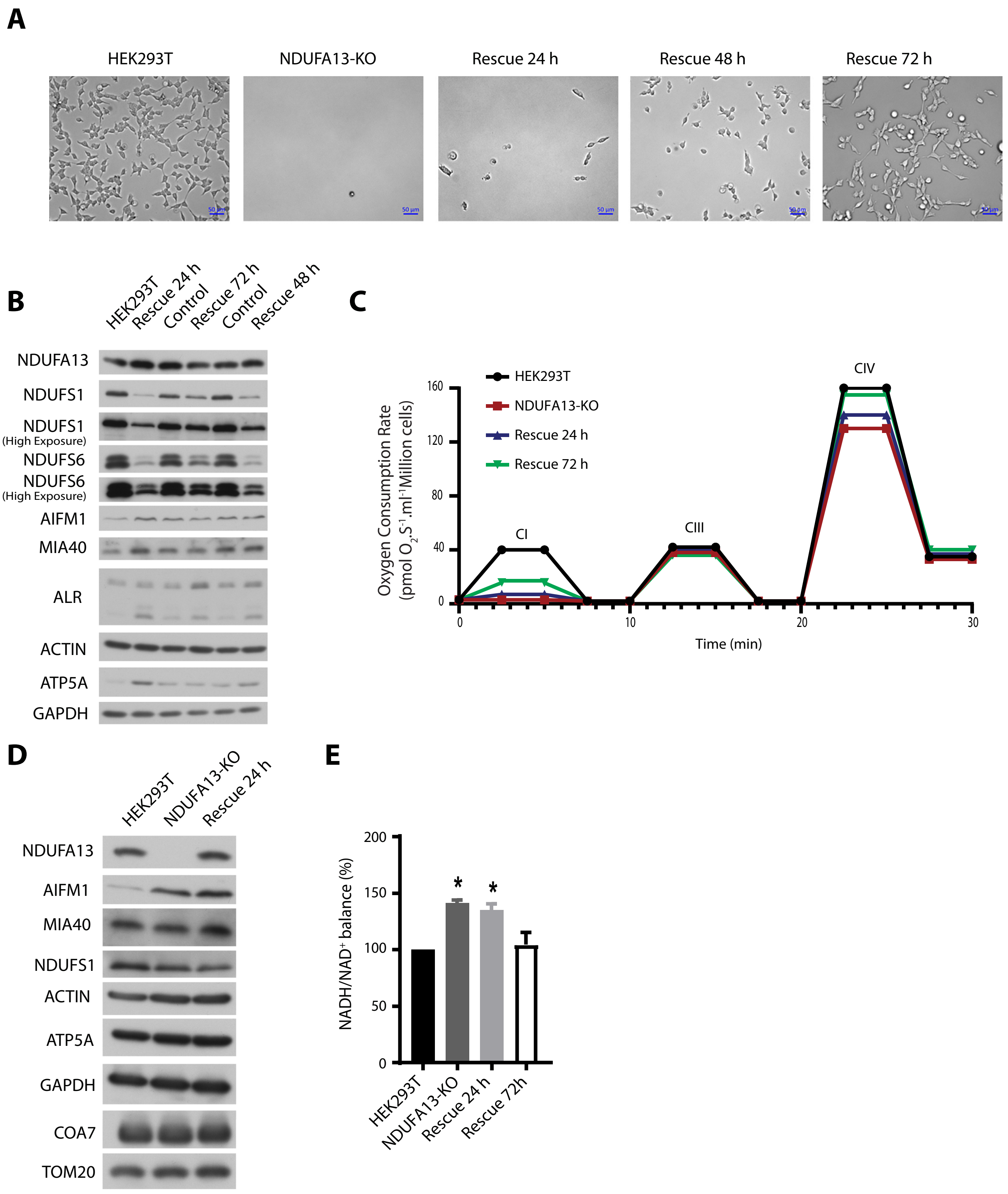
A
HEK293T
NDUFA13-KO
Rescue 24 h
Rescue 48 h
Rescue 72 h
B
HEK293T
Rescue 24 h
Control
Rescue 72 h
Control
Rescue 48 h
NDUFA13
NDUFS1
NDUFS1 (High Exposure)
NDUFS6
NDUFS6 (High Exposure)
AIFM1
MIA40
ALR
ACTIN
ATP5A
GAPDH
C
HEK293T
NDUFA13-KO
Rescue 24 h
Rescue 72 h
CI
CIII
CIV
Oxygen Consumption Rate (pmol O2.S-1.ml-1Million cells)
Time (min)
D
HEK293T
NDUFA13-KO
Rescue 24 h
NDUFA13
AIFM1
MIA40
NDUFS1
ACTIN
ATP5A
GAPDH
COA7
TOM20
E
NADH/NAD+ balance (%)
HEK293T
NDUFA13-KO
Rescue 24 h
Rescue 72h

 © The Author(s)

Figure EV3. Rescue of complex I activity in NDUFA13-KO cells.

(**A**) Contrast microscopy capture of the control HEK293T cells, complex I accessory subunit NDUFA13-KO (NDUFA13KO), and overexpression of NDUFA13 by plasmid transfection in NDUFA13-KO cells after 24 h (Rescue 24 h), 48 h (Rescue 48 h), or 72 h (Rescue 72 h). Cells were cultured and transfected in high-glucose medium for indicated time points, and then shifted to galactose medium for 24 h. (**B**) Cellular protein extracts were isolated from the control HEK293T cells, Rescue 24, 48, and 72 h. Cells were cultured and transfected in high-glucose medium for indicated time points, and then shifted to galactose medium for 24 h. The samples were analyzed by reducing SDS–PAGE and Western blot. (**C**) High-resolution respirometry profile of control HEK293T, NDUFA13-KO, Rescue 24 and 72 h performed in high-glucose. (**D**) Cellular protein extracts were isolated from control HEK293T, NDUFA13-KO, and overexpression of NDUFA13 by plasmid transfection in NDUFA13-KO cells after 24 h in high-glucose. The samples were analyzed by reducing SDS–PAGE and Western blot. (**E**) NADH/NAD^+ balance was measured in the control HEK293T, NDUFA13-KO, Rescue 24 h and 72 h. Data shown are mean ± SEM ($n = 3$ biological replicates). Statistical significance was obtained by an ordinary one-way ANOVA with Bonferroni multiple comparisons test (compared to HEK293T: NDUFA13-KO, $p = 0.0001$; Rescue 24 h, $p = 0.0004$; Rescue 72 h, $p > 0.9999$). *$p < 0.05$ indicates a significant difference from HEK293T cells.

© The Author(s)

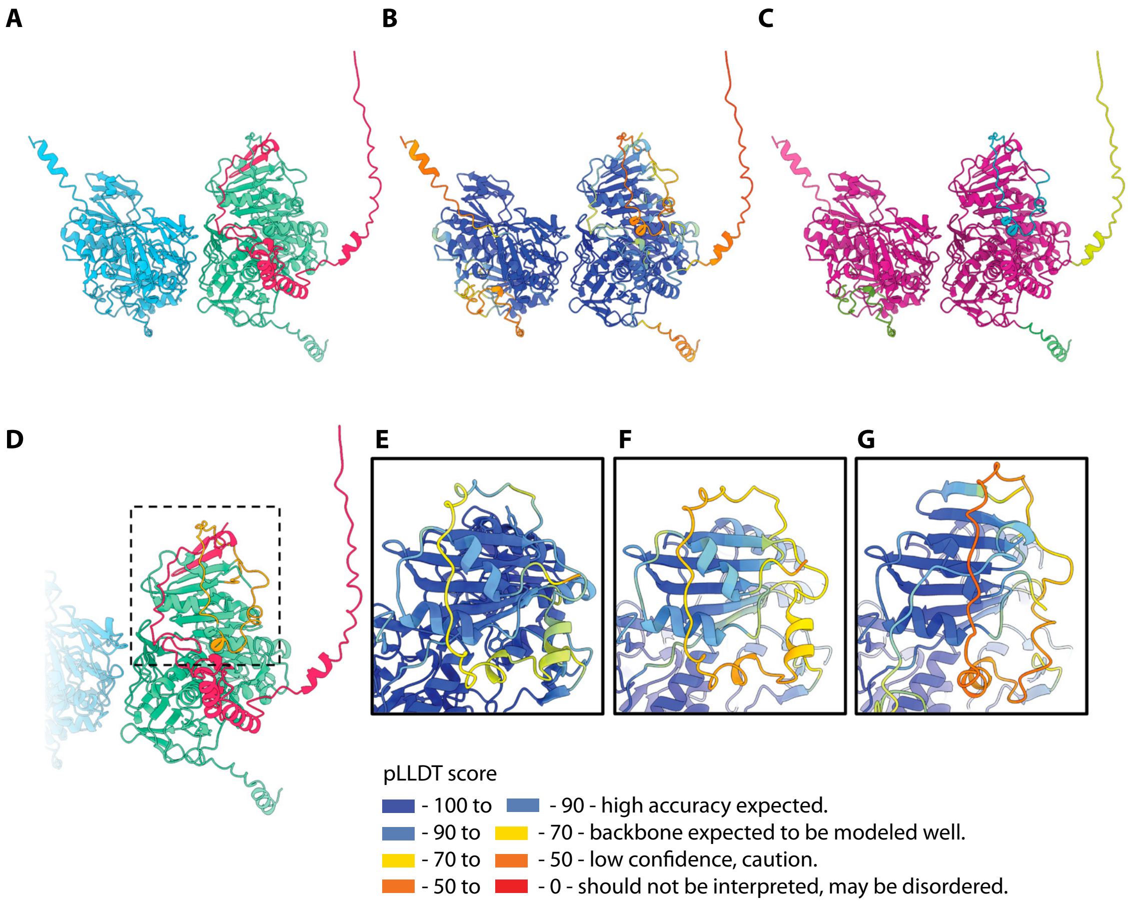

Figure EV4. Accuracy of the predicted structure of the MIA40-AIFM1 dimer complex.

The predicted structure of human MIA40-AIFM1 dimer complex is colored by (**A**), the chains composing the complex, (**B**) the AlphaFold per residue prediction confidence (pLLDT scores), (**C**) the Predicted Aligned Error (PAE), which reflects the confidence in the relative positions and orientations of parts of the predicted structure. Each color in (**C**) represents a coherent structural part of the complex, while the relative position of the parts highlighted by different colors remains ambiguous. (**D**) The overall structure of the MIA40-AIFM1 dimer with the region showed in the following panels indicated by the dashed line. The AlphaFold pLLDT scores for the AIFM1 C-loop showed a decrease in the confidence of the prediction when compared the (**E**), monomeric with (**F**), the dimeric form of the protein, indicating that the region is more likely to be unstructured when the AIFM1 dimerizes. (**G**) Further decrease of AIFM1 C-loop prediction quality was observed upon MIA40 binding.

 © The Author(s)

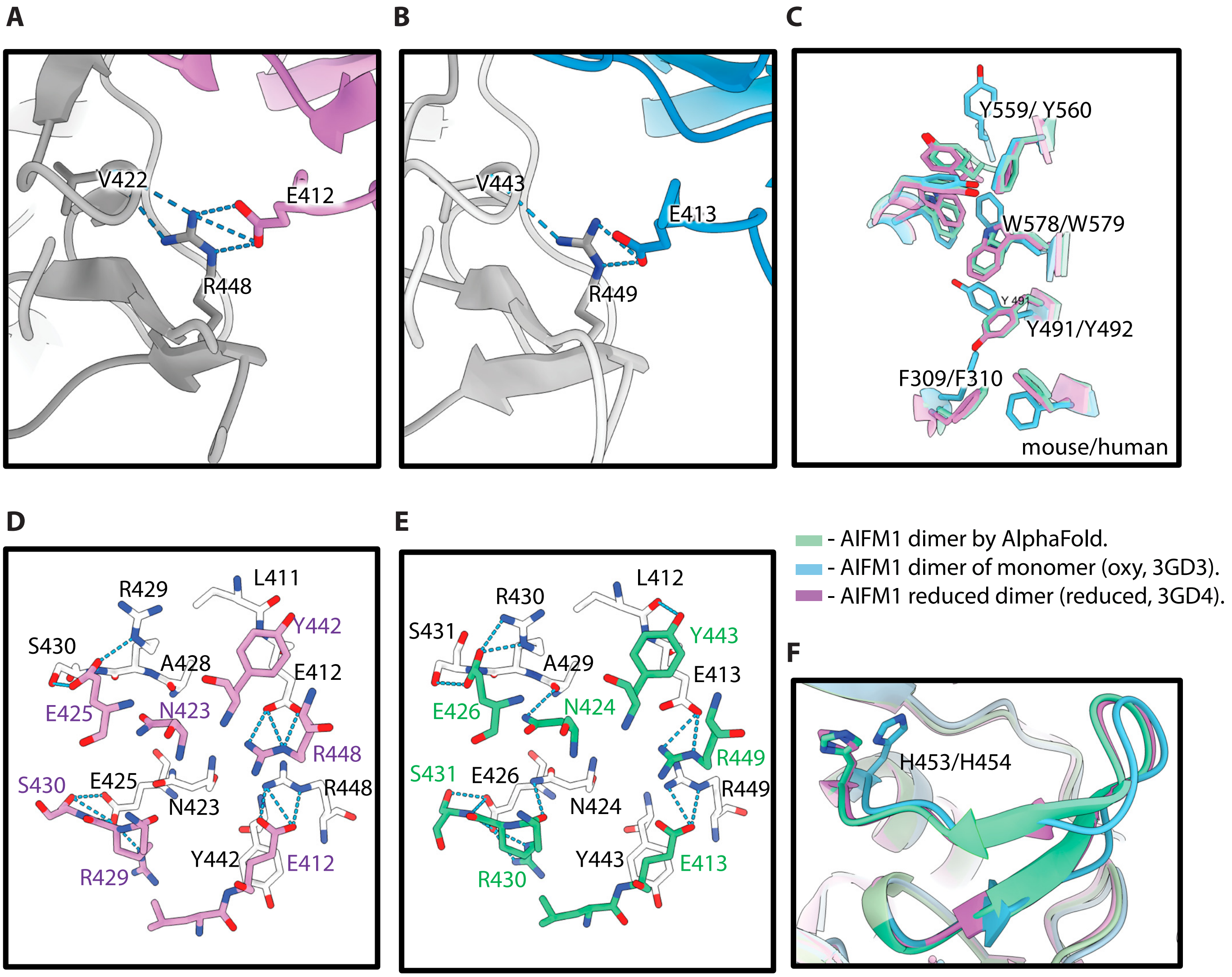

Figure EV5. Comparison of the AIFM1 dimer predicted and experimentally determined structures of the AIFM1 dimer.

(**A**) Hydrogen bond network formed by Glu412 and Arg448 in the naturally folded mouse dimer, and (**B**) the orthologous site formed by Glu413 and Arg449 in the predicted human AIFM1 dimer. (**C**) Side chains of residues that form the AIFM1 electron transfer chain analyzed in the crystal structures of mouse AIFM1 in oxidized monomeric (blue cartoon), its reduced dimeric form (pink cartoon), and the predicted human AIFM1 dimer (green cartoon). Residue numbers are provided for both orthologs of the protein (muse/human). The hydrogen bond network and conformation of the side chains at the dimerization interface were compared between (**D**), the naturally folded AIFM1 dimer, and (**E**), the predicted dimer. (**F**) The His454 (His453 in the mouse ortholog) side chain conformation was compared in the crystal structures of mouse oxidized monomeric AIFM1, its reduced dimeric form, and the predicted human AIFM1 dimer (color scheme same as in **C**).

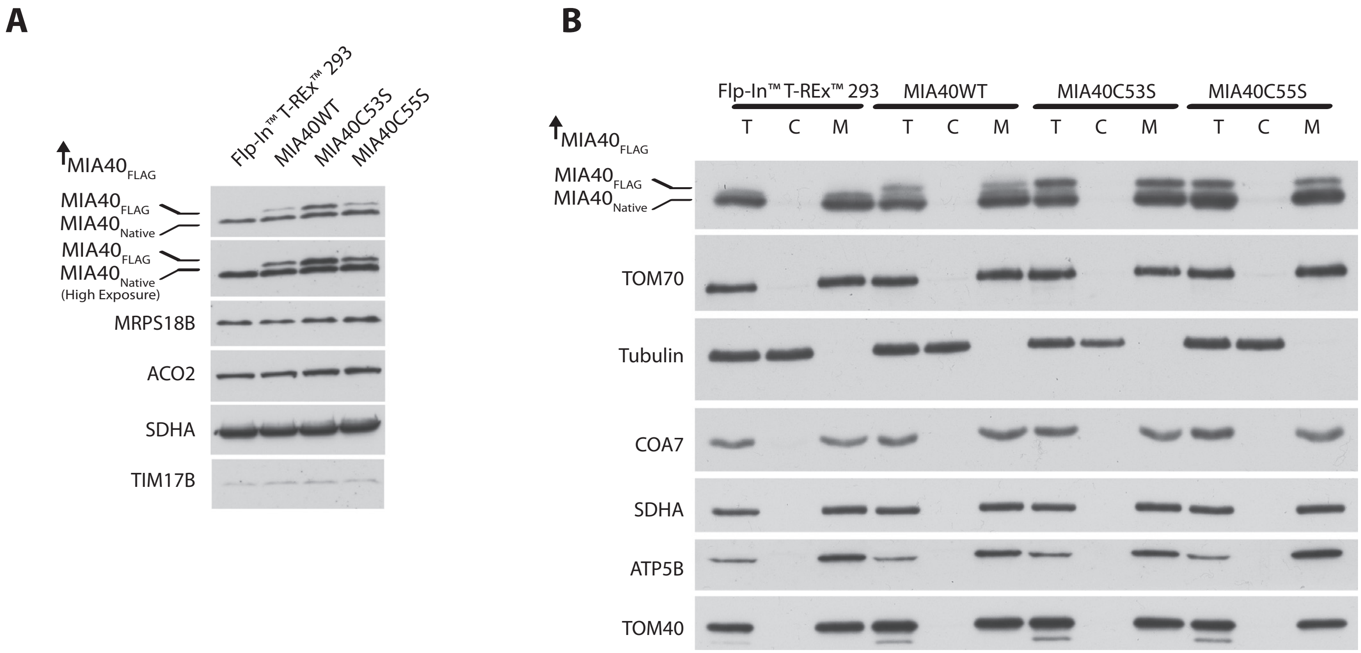

Figure EV6. Protein levels and localization of MIA40 variants.

(**A**) Total protein levels and (**B**) subcellular fractionation of Flp-In T-REx293 cells with induced expression of wild-type or MIA40$_{FLAG}$ variants C53S or C55S (MIA40WT, MIA40C53S or MIA40C55S, respectively). Total post-nuclear supernatant (T), cytosol (C), and mitochondria (M). The samples were analyzed by SDS–PAGE and Western blot.

 © The Author(s)

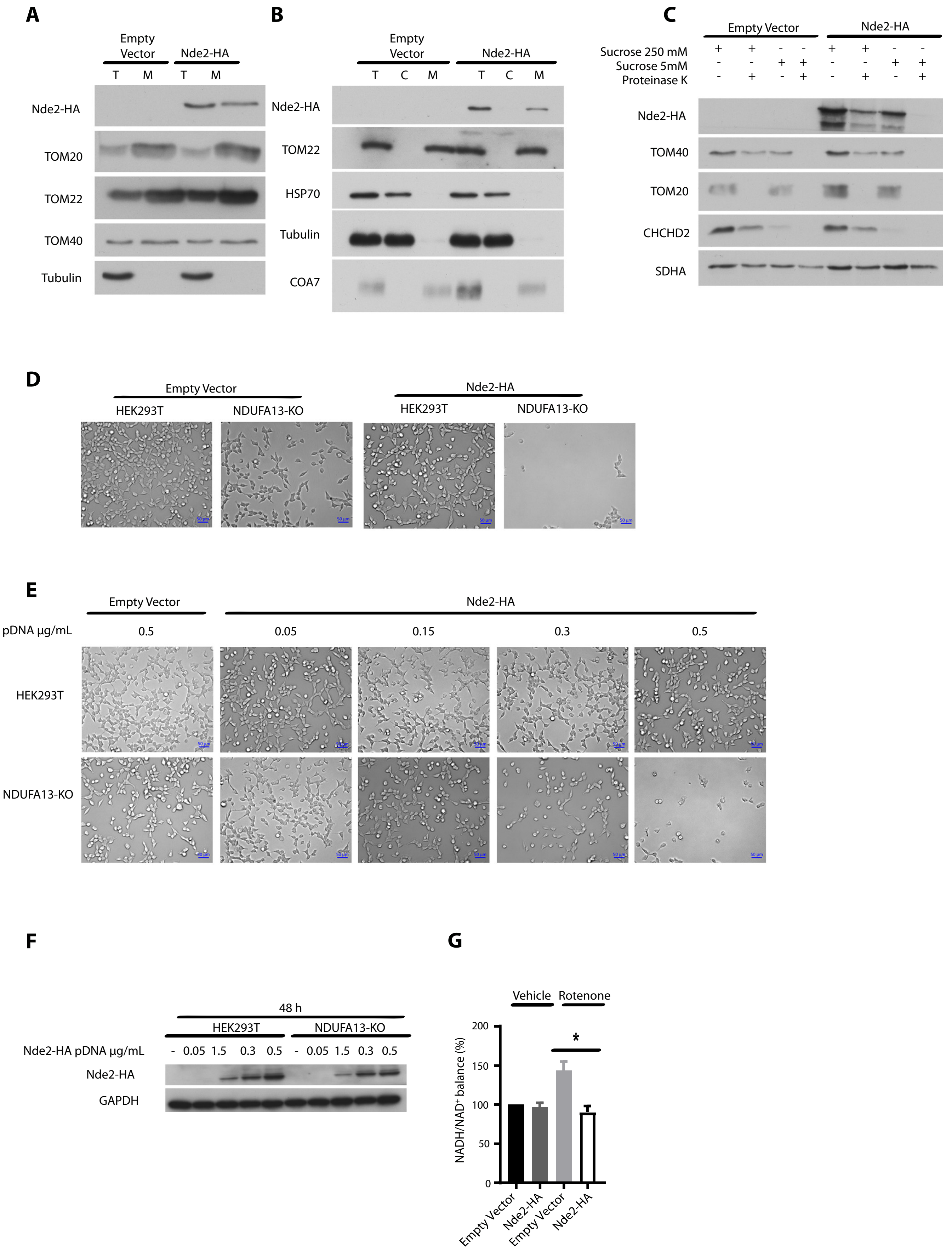
A
Empty Vector
Nde2-HA
T M T M
Nde2-HA
TOM20
TOM22
TOM40
Tubulin
B
Empty Vector
Nde2-HA
T C M T C M
Nde2-HA
TOM22
HSP70
Tubulin
COA7
C
Empty Vector
Nde2-HA
Sucrose 250 mM + + - - + + - -
Sucrose 5mM - - + + - - + +
Proteinase K - + - + - + - +
Nde2-HA
TOM40
TOM20
CHCHD2
SDHA
D
Empty Vector
Nde2-HA
HEK293T
NDUFA13-KO
HEK293T
NDUFA13-KO
E
Empty Vector
Nde2-HA
pDNA µg/mL
0.5
0.05
0.15
0.3
0.5
HEK293T
NDUFA13-KO
F
48 h
HEK293T
NDUFA13-KO
Nde2-HA pDNA µg/mL - 0.05 1.5 0.3 0.5 - 0.05 1.5 0.3 0.5
Nde2-HA
GAPDH
G
Vehicle
Rotenone
NADH/NAD+ balance (%)
200
150
100
50
0
*
Empty Vector
Nde2-HA
Empty Vector
Nde2-HA

© The Author(s)

Figure EV7. Topology and activity of external NADH-ubiquinone oxidoreductase 2 tagged with HA at C-terminus (Nde2-HA).

(**A**) Subcellular localization of Nde2-HA upon its transfection in the HEK293T cells. Cellular protein extracts (T) or isolated mitochondria (M) of HEK293T cells transfected with an empty plasmid or Nde2-HA were analyzed by reducing SDS–PAGE and Western blot. (**B**) subcellular fractionation of the HEK293T cells transfected with an empty vector or Nde2-HA. Total post-nuclear supernatant (T), cytosol (C), and mitochondria (M). The samples were analyzed by SDS–PAGE and Western blot. (**C**) Submitochondrial localization of Nde2-HA upon its transfection in the HEK293T cells analyzed by limited degradation by proteinase K in intact mitochondria (250 mM sucrose) or mitoplasts (5 mM sucrose). The samples were analyzed by SDS–PAGE and Western blot. (**D**) Contrast microscopy of HEK293T and complex I accessory subunit NDUFA13-KO transfected with an empty plasmid or Nde2-HA. (**E**) Contrast microscopy images of HEK293T and NDUFA13-KO cells transfected with increasing concentrations of the Nde2-HA plasmid after 48 h. (**F**) Cellular protein extracts were isolated from HEK293T and NDUFA13-KO cells that were transfected with different concentrations of Nde2-HA plasmid for 48 h. The samples were analyzed by reducing SDS–PAGE and Western blot. (**G**) HEK293T cells were transfected with an empty vector or Nde2-HA for 48 h. Then, the cells were incubated with a vehicle or 10 nM rotenone for 12 h. Afterward, the measurements of NADH/NAD^+ balance were performed. Data shown are mean ± SEM ($n = 3$ biological replicates). Statistical significance was obtained by an ordinary one-way ANOVA with Bonferroni multiple comparisons test (compared to HEK293T; HEK293T + Nde2-HA, $p > 0.9999$; HEK293T + rotenone, $p = 0.0003$; HEK293T + Nde2-HA + rotenone, $p = 0.4753$). *$p < 0.05$ indicates a significant difference from HEK293T cells treated with rotenone.

 © The Author(s)
